# Genetic Patterns Related with the Development and Progression of Sarcopenia and Sarcopenic Obesity: A Systematic Review

**DOI:** 10.3390/medicina61050866

**Published:** 2025-05-08

**Authors:** Andreea-Dalila Nedelcu, Andreea-Bianca Uzun, Viorela-Mihaela Ciortea, Laszlo Irsay, Liliana-Elena Stanciu, Dan Marcel Iliescu, Florina Ligia Popa, Mădălina-Gabriela Iliescu

**Affiliations:** 1 Faculty of Medicine, Doctoral School, “Ovidius” University of Constanta, 1 University Alley, Campus-Corp B, 900470 Constanta, Romania; dalila.nedelcu@365.univ-ovidius.ro (A.-D.N.); bianca.uzun@365.univ-ovidius.ro (A.-B.U.); liliana.stanciu@365.univ-ovidius.ro (L.-E.S.); 2 Faculty of Medicine, “Ovidius” University of Constanta, 1 University Alley, Campus-Corp B, 900470 Constanta, Romania; dan.iliescu@365.univ-ovidius.ro; 3 Department of Rehabilitation Medicine, University of Medicine and Pharmacy “Iuliu Hatieganu”, 8 Victor Babes Street, 400012 Cluj-Napoca, Romania; viorela.ciortea@umfcluj.ro (V.-M.C.); laszlo.irsay@umfcluj.ro (L.I.); 4 Physical Medicine and Rehabilitation Department, Faculty of Medicine, “Lucian Blaga” University of Sibiu, Victoriei Blvd., 550024 Sibiu, Romania; florina-ligia.popa@ulbsibiu.ro

**Keywords:** sarcopenia, sarcopenic obesity, single-nucleotide polymorphisms, SNPs

## Abstract

*Background and Objectives:* Despite their high prevalence, sarcopenia and sarcopenic obesity remain underdiagnosed worldwide, significantly impacting the health and quality of life of aging individuals. Due to their multifactorial nature, the current management strategies do not address their underlying pathogenesis. This systematic review aims to identify single-nucleotide polymorphisms (SNPs) associated with sarcopenia and/or sarcopenic obesity in humans. *Materials and Methods*. This systematic literature review followed the “Preferred Reporting Items for Systematic Reviews and Meta-Analyses (PRISMA)” guidelines and the protocol registered in PROSPERO. Extensive research was performed in six databases (PubMed, Web of Science, Cochrane Library, Scopus, ScienceDirect, and SpringerLink) using keywords such as “sarcopenia”, “sarcopenic obesity”, “single nucleotide polymorphisms”, “SNPs”, and “genetic variants”. The Q-Genie and ROBINS-E tools were utilized to assess the quality of the included studies. *Results:* The final analysis included 12 studies, which were classified as good-quality according to the Q-Genie assessment and indicated a low to moderate risk of bias according to the ROBINS-E evaluation, collectively identifying 43 SNPs significantly associated with sarcopenia or sarcopenic obesity. Specifically, 24 SNPs were linked to sarcopenia, while 19 were associated with sarcopenic obesity. *Conclusions:* Understanding the implications of SNPs provides valuable insights into individual susceptibility and the variability observed across populations, potentially leading to more targeted and effective diagnostic and treatment strategies. Advancing clinical practice requires ongoing research into the genetic aspects of sarcopenia and sarcopenic obesity.

## 1. Introduction

An increasing proportion of elderly individuals within the global population is one of the most defining demographic trends of the 21st century [1]. Over the past five decades, human life expectancy has risen significantly, marking a major public health achievement. However, this progress has also introduced substantial socio-demographic challenges, driven by the rapid aging of the population and the resulting surge in the prevalence of chronic disease [2]. The disparity between individuals who experience healthy aging and those who undergo an accelerated decline remains largely unknown [3]. Given that age-related changes in body composition contribute to functional decline and adverse health outcomes, addressing these challenges has become increasingly important [1]. While numerous aspects of aging are influenced by genetic factors, the specific genes involved have yet to be fully identified [3].

Sarcopenia is a common condition characterized by the gradual loss of muscle mass, accompanied by a decline in muscle strength and physical performance as part of the aging process [4,5]. The early detection of sarcopenia is essential, as regaining the skeletal muscle mass that has already been lost is extremely difficult [6]. Sarcopenia has been gaining significant research interest, with continuous efforts to convert the current knowledge of its pathophysiology into enhanced diagnosis and treatment strategies [4].

Sarcopenic obesity is defined as the coexistence of sarcopenia and obesity in an individual, characterized by the concurrent accumulation of body fat and the loss of muscle mass [7,8]. These changes contribute to a decline in physical performance, increased frailty, and an increased risk of mortality [9,10]. Therefore, sarcopenic obesity may lead to significantly more severe health consequences than either obesity or sarcopenia alone [11].

Despite their high prevalence, sarcopenia and sarcopenic obesity remain underdiagnosed worldwide, significantly impacting health and quality of life in aging individuals. Diagnosing these conditions is contentious and challenging because their symptoms are often masked by coexisting comorbidities common in elderly patients.

The development of sarcopenia and sarcopenic obesity is complex and influenced by both genetic and environmental factors [12,13]. Even under similar biological and environmental conditions, there is considerable individual variation in the quantity and quality of skeletal muscle [14,15]. Also, the variation in the occurrence of sarcopenia among elderly individuals of the same age [16] suggests that certain individuals exhibit a greater predisposition to developing this condition. These observations underscore the significance of investigating the genetic factors that contribute to its pathophysiology, as they may serve as key determinants of the interindividual variability observed.

Due to the multifactorial nature of these conditions [11,17,18], their management is not currently based on their underlying pathogenesis. The early detection of individual susceptibility through simple genetic testing could enable timely interventions, such as personalized physical activity programs, while a deeper understanding of the genetic factors and biological mechanisms underlying sarcopenia and sarcopenic obesity may pave the way for more targeted therapeutic strategies to improve their prognosis.

Certain single-nucleotide polymorphisms (SNPs) may play a significant role in increasing individual susceptibility to sarcopenia or sarcopenic obesity. Understanding these genetic variations is important for identifying at-risk individuals and developing prevention or therapeutic individualized intervention strategies. This systematic review aimed to identify, analyze, and synthesize SNPs that are relevant to sarcopenia and sarcopenic obesity in humans.

By systematically collecting and analyzing data from multiple studies, we aimed to increase the effective sample size, improve the robustness of the findings, and enhance the reliability of the conclusions. Additionally, the systematic review approach facilitated the identification of genetic patterns across diverse populations and emphasized gaps in the current knowledge that require further investigation.

## 2. Materials and Methods

This systematic review was reported in accordance with the PRISMA guidelines, which are internationally recognized for standardizing the reporting of systematic reviews [19]. These guidelines ensure the transparent presentation of the rationale for the review, the methodology applied, and the results obtained [19].

Additionally, the protocol used for this review was submitted to PROSPERO (International Prospective Register of Systematic Reviews) under protocol registration number CRD420251007921.

### 2.1. The Search Strategy

The systematic review was conducted through an extensive search conducted until March 2025 across six databases, including PubMed, National Institutes of Health (NIH); Web of Science; Cochrane Library; Scopus; ScienceDirect; and SpringerLink Journals. To optimize the selection, filters were applied to restrict the publication period to 2015–2025, including only studies in English and focusing on human subjects.

The search strategy was designed using relevant terms such as ”sarcopenia”, ”sarcopenic obesity”, ”single nucleotide polymorphisms”, ”SNPs”, and ”genetic variants”, combined using the Boolean operators ”AND” and ”OR”. The final search phrase used was ”(sarcopenia OR sarcopenic obesity) AND (single nucleotide polymorphisms OR SNPs OR genetic variants)”.

This systematic search strategy ensured a broad yet targeted collection of the literature, supporting a rigorous review process. Further details regarding the search strategy and database-specific filters are presented in Table 1. The articles were collected and managed using Zotero to ensure better organization and efficiently identify and remove duplicates. The screening process was then conducted based on the title, abstract, and predefined inclusion and exclusion criteria, followed by a full-text analysis as part of the systematic review.

### 2.2. The Study Selection

Two reviewers independently conducted the screening process based on predefined inclusion and exclusion criteria. The process involved an initial screening of the titles and abstracts, followed by a full-text review of studies considered potentially eligible. Any discrepancies between the reviewers were resolved through discussion and consensus.

The Inclusion Criteria:

- Original studies (randomized controlled trials, case–control studies, cohort studies, observational studies, trials) investigating SNPs associated with a predisposition to or the progression of sarcopenia and/or sarcopenic obesity;
- The association of SNPs with muscle mass, muscle strength, muscle function, or body composition in the context of sarcopenia or sarcopenic obesity;
- Studies conducted on adults, particularly older individuals, diagnosed with sarcopenia or sarcopenic obesity based on universally accepted criteria;
- Studies published in English;
- Studies published in the last 10 years.

The Exclusion Criteria:

- Systematic reviews, meta-analyses, books, book chapters, editorials, conference abstracts, and notes;
- Studies not focused on SNPs associated with sarcopenia/sarcopenic obesity;
- Studies analyzing environmental or lifestyle factors involved in the pathogenesis of sarcopenia and sarcopenic obesity without investigating the genetic component;
- Studies examining the genetic context between sarcopenia/sarcopenic obesity and other pathologies;
- Studies that did not provide a clear analysis of the associations between SNPs and muscle mass, muscle strength, or body composition;
- Studies conducted on children, adolescents, or individuals with genetic disorders affecting muscle mass;
- Animal studies or preclinical studies without clinical validation;
- Studies published in languages other than English;
- Studies published before 2015, ensuring the relevance and up-to-date nature of the data.

### 2.3. Data Extraction

Data extraction was performed independently by three reviewers using a standardized data extraction form. Any discrepancies between reviewers were resolved through discussion and consensus.

### 2.4. Quality Assessment

The methodological quality of the included studies was assessed using two specific tools. The evaluation was performed independently by two reviewers, with discrepancies resolved through discussion or consultation with a third reviewer.

### 2.5. Statistical Analysis

Due to the heterogeneity of the study designs and outcome measures, a qualitative synthesis was primarily conducted. A formal meta-analysis was not performed due to the methodological variability across studies.

## 3. Results

**Figure 1:**
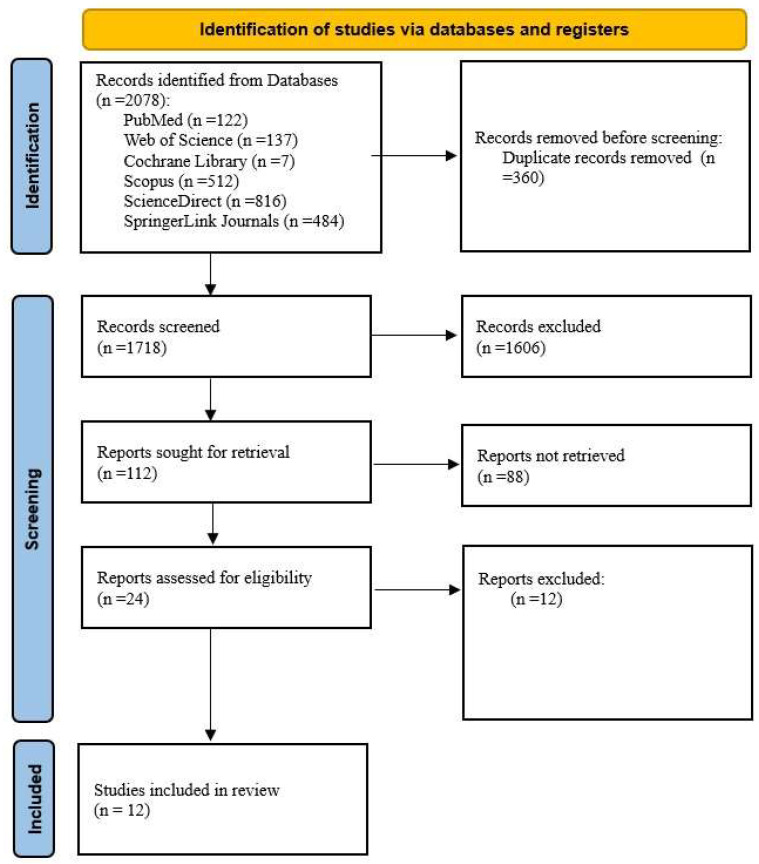
Adapted PRISMA flow diagram. Source: [19].

A total of 2078 studies were initially identified, as presented in Table 1. Among the databases explored, ScienceDirect yielded the highest number of results, with eight hundred and sixteen, followed by Scopus, which returned five hundred and twelve, and SpringerLink Journals, with four hundred and eighty-four. Web of Science contributed one hundred and thirty-seven results, while PubMed identified one hundred and twenty-two relevant studies. Cochrane Library provided a more limited number of studies, with only seven results. These identified studies formed the foundation for the subsequent screening and selection process. After the removal of duplicates, 1718 articles remained. Subsequent screening of their titles and abstracts resulted in the exclusion of 1606 studies. A full-text assessment was then conducted for the 112 remaining articles, leading to the inclusion of 24 studies (Figure 1).

### 3.1. The Included Studies

After assessing their eligibility, 12 studies were selected for inclusion in this review (Figure 1).

The studies included in this review are systematically organized within Table 2, which presents key details such as the author’s name and year of publication, country, pathology, study design, sample size, the number of SNPs identified, and references. The Q-Genie and ROBINS-E tools were utilized to assess the quality of the included studies [20,21].

### 3.2. The PICO Question

The included studies were selected based on their relevance to the central research question of this systematic review: “Which SNPs are associated with the predisposition to or progression of sarcopenia and/or sarcopenic obesity, according to existing studies?” This question is structured based on the following PICO components:

- P (Population): Individuals affected by sarcopenia or sarcopenic obesity, specifically adults, with a focus on the elderly;
- I (Intervention): The identification of SNPs associated with the development of sarcopenia and/or sarcopenic obesity;
- C (Comparison): Healthy control groups without sarcopenia/sarcopenic obesity or individuals with different SNPs to assess variations and differences;
- O (Outcome): The association between specific SNPs and the predisposition to or the progression of sarcopenia or sarcopenic obesity.

### 3.3. Risk of Bias

The evaluation of all of the studies using the Q-Genie tool determined scores that classified these articles as good-quality studies, indicating a well-structured methodology and reliable genetic associations. Q-Genie is a tool designed to assess the quality of these types of studies and is particularly useful for systematic reviews and meta-analyses, which are essential methods for synthesizing findings and estimating the impact of genetic variants on traits of interest. The tool exhibits excellent psychometric properties and assigns a quality score to each study, categorizing it as poor-, moderate-, or good-quality [20,21]. The validity of the included studies was assessed using this tool, as detailed in Table 3.

The ROBINS-E tool provides a systematic method for evaluating the risk of bias in observational epidemiological studies [34]. It can be a powerful resource for assessing the quality of genetic studies, ensuring that findings are not influenced by bias.

The ROBINS-E tool evaluates the risk of bias across seven key domains: D1: Risk of bias due to confounding; D2: Risk of bias arising from measurement of the exposure; D3: Risk of bias in selection of participants into the study (or into the analysis); D4: Risk of bias due to post-exposure interventions; D5: Risk of bias due to missing data; D6: Risk of bias arising from measurement of the outcome; and D7: Risk of bias in selection of the reported result [34]. Each domain is assessed by the reviewers for risk of bias and rated as low, some concerns, or high, based on the study’s design and methodology. Any discrepancies between reviewers were addressed to ensure consistency. These domain-level ratings are then aggregated to provide an overall risk of bias score.

**Figure 2:**
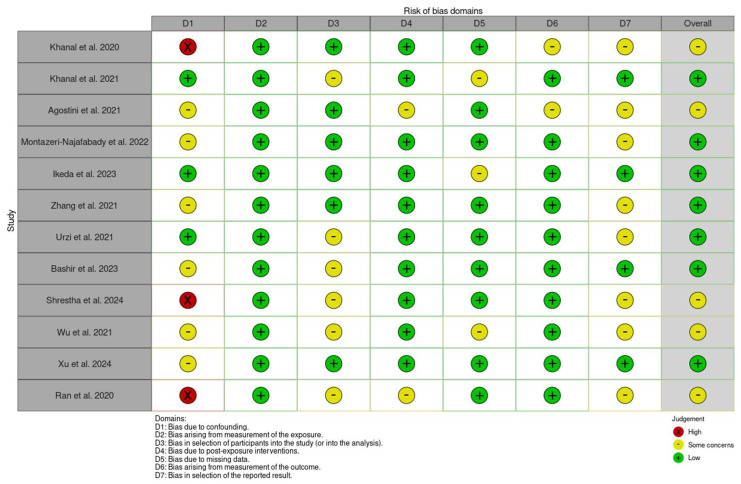
The risk of bias assessment using ROBINS-E for the included studies [1,2,22,23,24,25,26,27,29,30,31].

**Figure 3:**
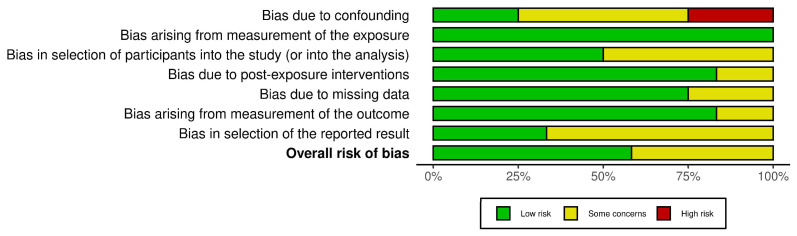
A summary plot of ROBINS-E for the included studies.

The studies included in this systematic review are of moderate- to high-quality, with a generally low risk of bias. While some studies require careful interpretation due to potential biases, the findings remain credible, as outlined in Figure 2 and Figure 3.

### 3.4. Detailed Study Descriptions of Identified SNPs

Centralized data from the studies’ descriptions are presented in Table 4. Recent advancements in genomic technology have significantly increased the number of published genetic studies. Khanal et al. [22], in a study published in 2020, highlighted the impact of the definitions of sarcopenia on the prevalence estimates and genetic associations in 307 Caucasian female participants over 60 years old. According to the definition of skeletal muscle mass percent (%SMM), individuals were classified as sarcopenic if their skeletal muscle mass comprised less than 22.1% of their total body weight. The skeletal muscle mass index (SMI) criterion classified individuals with a skeletal muscle mass index of 6.76 kg/m^2^ or lower as having sarcopenia. The European Working Group on Sarcopenia in Older People (EWGSOP) criteria diagnosed sarcopenia when both the SMI was below 6.76 kg/m^2^ and handgrip strength was measured as less than 20 kg. Depending on the definition used, the prevalence of sarcopenia ranged from 1.3% to 60.6%. The participants were classified into two groups: sarcopenic and non-sarcopenic. This study analyzed 24 SNPs but found that only 4 SNPs (FTO rs9939609, ESR1 rs4870044, NOS3 rs1799983, and TRHR rs7832552) were significantly associated with sarcopenia. The genotyping method used in said study was the Fluidigm employed as the primary system [22]. Also, Khanal et al. [23], in a study published in 2021, highlighted the relationship between sarcopenia, obesity, and genetic variants (SNPs) in 307 Caucasian female participants (aged 71 ± 6 years). Sarcopenia was defined by a low skeletal muscle mass index (SMI < 6.76 kg/m^2^) and reduced muscle strength (handgrip strength < 28.5 kg), while obesity was defined by a high body fat percentage (>38%). Participants were classified into four groups based on sarcopenia and obesity status: sarcopenic obese, non-sarcopenic obese, sarcopenic non-obese, and non-sarcopenic non-obese. The prevalence of sarcopenic obesity was 25.1%, the highest of the four groups. This study analyzed 24 SNPs but found that only ACTN3 rs1815739, MTHFR rs1801131, and MTHFR rs1537516 were found to be significantly associated with sarcopenia in obese women, highlighting an increased risk of developing this condition based on genetic profile. The genotyping method used in said study was the Fluidigm employed as the primary system [23].

Agostini et al. [2] investigated the role of the rs363050 polymorphism of the synaptosomal-associated protein of 25 kDa (SNAP-25) gene and the expression of certain circulating microribonucleic acid (miRNAs) associated with this gene in patients with sarcopenia in 2021. Additionally, they examined how a structured rehabilitation program influenced the expression of these miRNAs. Sarcopenia was assessed based on the EWGSOP criteria, including a low Short Physical Performance Battery (SPPB) score and a reduced handgrip strength. The genotypic analysis, performed using the TaqMan SNP Genotyping Assay on 358 patients (177 sarcopenic patients and 181 controls), revealed that the AA genotype of the rs363050 SNP is significantly more frequent in patients with sarcopenia (40% vs. 27% in controls), suggesting a genetic predisposition. One limitation of this study is that sarcopenia screening relied on physical performance and muscle strength indices without incorporating imaging parameters, which are considered the gold standard [2].

The study conducted by Montazeri Najafabady et al. [24] in 2022 explored the association between TP53 codon 72 (rs1042522, Arg72Pro) and intron 3 16-bp Del/Ins (rs17878362) polymorphisms and susceptibility to sarcopenia in older Iranian adults. The study population consisted of 254 Iranian adults aged 65 years or older, including 65 individuals diagnosed with sarcopenia and 189 healthy controls. Sarcopenia was defined based on the EWGSOP criteria. Muscle mass was assessed using a segmental multi-frequency bioelectrical impedance analysis (BIA). Low muscle mass was classified as an SMI < 7.0 kg/m^2^ for males and <5.7 kg/m^2^ for females. Muscle function was evaluated through handgrip strength (HGS) and gait speed (GS). HGS was measured as the mean of three trials for both hands, with a low muscle strength defined as <26 kg for males and <18 kg for females. Gait speed was assessed over a 4 m walk, with a speed of <0.8 m/s indicating low physical performance. Genotyping of polymorphisms was performed using the polymerase chain reaction–restriction fragment length polymorphism (PCR-RFLP) technique [24].

The study conducted by Ikeda et al. [25] in 2023 examined the association between the resistin G–A haplotype at SNP-420 (rs1862513) and SNP-358 (rs3219175) and latent sarcopenic obesity in a Japanese cohort. The study population consisted of 567 participants attending annual medical check-ups as part of the Toon Genome Study. Sarcopenic obesity was assessed using a latent sarcopenic obesity index, defined as a visceral fat area (VFA) ≥ 100 cm^2^ and at least one of the following: low muscle mass (SMI ≤ 7.4 kg/m^2^ in males, ≤5.8 kg/m^2^ in females), a weak grip strength (≤32.5 kg in males, ≤20.0 kg in females), or a slow physical performance (≥6.7 s duration in the Timed Up and Go test). Genotyping was performed using the TaqMan assay [25].

Zhang et al. [26] explored the genetic susceptibility to sarcopenia among Tibetans living at high altitudes in 2021. This research involved 1447 participants, including 438 individuals with sarcopenia and 1009 healthy controls. From this cohort, 160 individuals (80 men, 80 women; mean age: 53.19 years) were selected for the genetic analysis. Sarcopenia was diagnosed based on the Asian Working Group for Sarcopenia (AWGS) criteria, using the following thresholds: a skeletal muscle mass index (SMI) below 8.07 kg/m^2^ for men and 6.62 kg/m^2^ for women, a handgrip strength under 26.7 kg for men and 15.8 kg for women, and a gait speed below 0.8 m/s. Polymerase chain reaction (PCR) was used for SNP amplification, analyzing four single-nucleotide polymorphisms (SNPs): FTO rs9939609 and rs9936385, ACVR2B rs2276541, and IRS1 rs2943656 [26].

The study conducted by Urzi et al. [1] in 2021 explored the association between Methylenetetrahydrofolate reductase (MTHFR, rs1801131), Alpha-actinin-3 (ACTN3, rs1815739), and Nuclear respiratory factor 2 (NRF2, rs12594956) polymorphisms and the susceptibility to age-related sarcopenia in older Slovenian adults. The study population consisted of 190 older adults (67 men, 123 women) residing in nursing homes in southern Slovenia, including 45 individuals diagnosed with sarcopenia and 145 healthy controls. Sarcopenia was defined based on the EWGSOP criteria, with their muscle mass assessed through a bioelectrical impedance analysis (BIA). A low muscle mass was classified as an SMI < 8.87 kg/m^2^ for males and <6.42 kg/m^2^ for females. Muscle function was evaluated using handgrip strength and gait speed, with low strength defined as <27 kg for men and <16 kg for women and low gait speed as <0.8 m/s for both. Genotyping of polymorphisms was performed using the KASP assay, a competitive allele-specific PCR method [1].

The study conducted by Bashir et al. [27] in 2023 investigated the association between activin type I receptor polymorphisms (rs10783486 and rs2854464) and body composition in older individuals with sarcopenia, using data from the LACE randomized controlled trial, which investigated the effects of the Angiotensin-Converting Enzyme (ACE) inhibitor Perindopril and/or leucine on physical performance and muscle mass in older adults with sarcopenia [32]. This study included 110 participants aged 70 years and older who were all diagnosed with sarcopenia according to the EWGSOP criteria. Body composition was assessed using dual-energy X-ray absorptiometry (DXA), while muscle strength was evaluated through handgrip strength and maximal voluntary contraction of the quadriceps. Genotyping of the ACVR1B polymorphisms was performed using TaqMan qPCR assays [27].

The study conducted by Shrestha et al. [29] in 2024 explored the association between BDKRB2 polymorphisms (rs1799722 and rs5810761) and physical performance and muscle mass in older adults with sarcopenia. This study consisted of 136 Caucasian patients with sarcopenia (mean age: 76.5 ± 5.5 years; 72 females and 64 males), recruited as part of the LACE randomized controlled trial [23]. Their skeletal muscle mass was assessed using a bioimpedance analysis, while their muscle strength was evaluated through handgrip strength (HGS) measurements utilizing a Jamar dynamometer, with predefined thresholds of <26 kg for males and <18 kg for females. Physical performance was assessed using gait speed and functional mobility tests, including the Short Physical Performance Battery (SPPB) and Six-Minute Walk Distance (6MWD). Gait speed was measured over a standardized 4 m walking test, with a velocity of <0.8 m/s. All assessments were conducted at the baseline, 6 months, and 12 months to evaluate longitudinal changes in muscle function. Genotyping of rs1799722 and rs5810761 was performed using the TaqMan and Hotstar Taq PCR methods [28].

The study conducted by Wu and Chen [30] in 2021 investigated the genetic associations with sarcopenia in an elderly Taiwanese population through a genome-wide association study (GWAS). This study recruited 96 participants aged 60 years or older. Sarcopenia was diagnosed based on the criteria from the Asian Working Group for Sarcopenia (AWGS). Their muscle mass was assessed using a bioelectrical impedance analysis (BIA), with an SMI < 7.0 kg/m^2^ for males and <5.7 kg/m^2^ for females indicating low muscle mass. Muscle function was evaluated through handgrip strength (HGS) and gait speed (GS). Low muscle strength was defined as an HGS < 28 kg for males and <18 kg for females, while a low physical performance was defined as a GS < 1.0 m/s. Genotyping was performed using the Affymetrix Axiom Genome-Wide TWB 2.0 array [30].

The study conducted by Xu et al. [31] in 2024 investigated the genetic associations with sarcopenic obesity (SO) in a large cohort from the UK Biobank using an exome-wide sequencing approach. Their analysis included 2887 SO cases and 113,284 controls in the sequenced dataset and 4003 SO cases with 161,990 controls in the imputed dataset. SO was defined based on the European Society for Clinical Nutrition and Metabolism (ESPEN) and the European Association for the Study of Obesity (EASO) consensus. Muscle function was assessed using handgrip strength (HGS), while body composition was evaluated through fat mass percentage (FM%) and appendicular lean mass adjusted for weight (ALM/W). Low muscle strength was defined as an HGS < 27 kg for males and <16 kg for females, whereas high adiposity was defined as an FM% > 27.3% for males and >40.7% for females. Genotyping was performed using the Illumina NovaSeq 6000 platform [31].

The study conducted by Shu Ran et al. [32] in 2020 investigated the genetic associations with sarcopenia in a Han Chinese population through a whole-exome sequencing (WES) and genome-wide association study (GWAS) approach. This study recruited 101 Chinese adults for the discovery phase, while a replication cohort included 217,822 individuals from the UK Biobank. Sarcopenia risk was assessed using whole lean body mass (WLBM), which was measured using dual-energy X-ray absorptiometry (DXA) in the Chinese cohort and a bioelectrical impedance analysis (BIA) in the UK Biobank cohort. This study aimed to identify single-nucleotide polymorphisms (SNPs) associated with WLBM variations [32].

## 4. Discussion

The analyzed studies were conducted across diverse populations, with the United Kingdom being the most represented, accounting for four studies. China followed closely, with four studies focusing on its population. Additionally, individual studies were carried out in Italy, Iran, Japan, and Slovenia, each contributing valuable insights into the genetic variations associated with sarcopenia and sarcopenic obesity. This geographical diversity enhances the generalizability of the findings by capturing the genetic differences across distinct ethnic backgrounds.

Utilizing various genotyping techniques, researchers collectively identified 43 single-nucleotide polymorphisms significantly associated with sarcopenia or sarcopenic obesity. Specifically, 24 SNPs were linked to sarcopenia, while 19 SNPs were associated with sarcopenic obesity. These SNPs were mapped to multiple genes, shedding light on potential genetic pathways underlying the pathophysiology of these conditions.

The substantial variation in the sarcopenia prevalence reported is largely attributed to inconsistencies in the definitions and threshold values [35,36]. These discrepancies may have significant implications for the ability to identify causal factors [22]. Standardizing the definitions and adopting polygenic risk scores could enhance future research and clinical strategies [22].

### 4.1. The Functional Pathways of the Specific SNPs

The SNPs identified in the studies included in this review were classified according to their functional pathways, and systematically grouping these variants enabled a clearer analysis of their relevance to sarcopenia and/or sarcopenic obesity (Supplementary Materials).

#### 4.1.1. Muscle Structure, Function, and Atrophy

The ACTN3 gene encodes the alpha-actinin-3 protein, which is exclusively expressed in fast-twitch type II muscle fibers [1]. Urzi et al. reported that individuals with the XX genotype of the ACTN3 rs1815739 polymorphism had twice the risk of developing sarcopenia (OR = 2.25), with the X allele being significantly overrepresented among sarcopenic individuals [1]. Furthermore, Khanal et al. reported that individuals carrying the ACTN3 rs1815739 CC genotype had 1.8 times higher odds of being classified as sarcopenic compared to T-allele carriers in an obese population [23]. This finding suggests a significant link between ACTN3 expression and muscle mass degradation in the context of obesity [23]. However, the current evidence regarding the role of ACTN3 rs1815739 in sarcopenia and sarcopenic obesity remains preliminary, limited by modest effect sizes, methodological constraints, and an insufficient mechanistic understanding. Advancing towards its clinical application will require polygenic risk models, functional validation, and genotype-guided intervention studies.

Genetic polymorphisms in key pathways involved in muscle regulation, such as the activin/myostatin signaling pathway, are thought to contribute to the development of sarcopenia [27]. Bashir et al. reported that polymorphisms in the activin IB receptor locus (ACVR1B rs2854464 and rs10783486) are associated with height and limb fat mass, rather than muscle mass or strength, in older men with sarcopenia [27]. These findings may have clinical relevance, as interventions targeting the activin/myostatin pathway could offer beneficial effects on extramuscular fat, reducing its impact on the physiology of the skeletal muscle [27]. These associations require further validation in larger and more diverse populations due to this study’s limitations, including a small sample size and the absence of a control group without sarcopenia [27].

While many SNPs are associated with an increased risk of sarcopenia, some studies have shown that certain genetic variants exert protective effects in different populations [24], underscoring the need for further research in diverse cohorts to fully understand their impact.

The TP53 gene encodes a 53 kDa protein that plays a role in multiple aspects of skeletal muscle cell function, including differentiation and overall physiology [24]. TP53 codon 72 (rs1042522) significantly influences sarcopenia risk, with Arg/Arg carriers being at higher risk, while the Arg/Pro and Pro/Pro genotypes exhibit a protective effect, reducing the likelihood of sarcopenia [24]. On the other hand, no significant association was found between intron 3 TP53 polymorphisms and sarcopenia susceptibility [24]. However, this polymorphism was observed to affect certain biochemical and metabolic parameters, suggesting its potential role in metabolic regulation [24]. Despite its findings, this study presents certain limitations, including a relatively small sample size and a geographically restricted selection of participants from southern Iran, which may affect the generalizability of the results to broader populations [24]. Clinical translation remains dependent on replication of the results and investigation of the underlying biological mechanisms.

#### 4.1.2. Neurotransmission

SNAP-25 is a key protein required for preserving both the structural and functional integrity of the neuromuscular junctions [2]. Agostini et al. reported that SNAP-25 rs363050 is associated with sarcopenia, and related miRNAs may function as both diagnostic biomarkers and indicators of the effectiveness of rehabilitation [2]. This highlights the significant role of genetic and epigenetic factors in the pathophysiology of sarcopenia [2]. This study identifies promising associations, but methodological limitations (the evaluation methods, small sample sizes, and insufficient functional validation) reduce the strength of its evidence. Clinical translation is possible but requires further studies. Additionally, this polymorphism has been associated with other age-related conditions, such as Alzheimer’s disease and type 2 diabetes [37,38].

#### 4.1.3. Lipid Metabolism and Adipogenesis

Recent studies have demonstrated a complex association between polymorphisms in the FTO gene (fat mass and obesity-associated gene) and the risk of sarcopenia, influenced by population differences, varying diagnostic criteria, and environmental factors [22].

Khanal et al. [22], in a study conducted in 2020, reported that using the %SMM definition, the FTO rs9939609 variant emerged as the strongest genetic risk factor. Individuals carrying the AA genotype were found to have a 3.04 times greater risk of sarcopenia compared to those with the T allele, suggesting the significant impact of this gene on muscle mass regulation. While FTO is widely recognized for its role in obesity and energy metabolism, these findings highlight its independent association with a lower skeletal muscle mass and sarcopenia [22]. Notably, the cohort consisted exclusively of Caucasian female participants. The observed effect sizes were modest and accompanied by wide confidence intervals. Although this SNP suggests potential for genetic risk stratification, its clinical applicability requires replication of the results in more diverse populations.

Zhang et al. [26] reported in 2021 that FTO rs9939609 and rs9936385 were significantly associated with lower limb skeletal muscle mass and sarcopenia, particularly in Tibetan women. Individuals with the TT genotype at rs9939609 had a higher risk of sarcopenia compared to that in A-allele carriers [26]. Although these results suggest their potential for genetic risk stratification and clinical translation, future studies should prioritize functional validation, replication in ethnically and sexually diverse cohorts, and longitudinal designs to confirm the biological relevance and clinical applicability of these variants.

Genome-wide association studies (GWASs) analyze genetic variations across the entire genome in large populations to uncover links between genotypes and phenotypes. Over the past decade, GWASs have transformed the study of complex disease genetics, revealing numerous significant associations with human traits and diseases [39].

The study conducted by Wu et al. [30] in 2021 explored the genetic associations with sarcopenia using a genome-wide association study. This study introduced novel genetic markers for sarcopenia, but its limitations included a small sample size and a restricted Taiwanese cohort, which may have impacted the generalizability of its findings [30]. The statistical analyses identified 12 single-nucleotide polymorphisms (SNPs) significantly associated with sarcopenia, with 8 of them being linked to more than one sarcopenic index (listed in Table 4). Notably, OSBPL3 rs10282247, which influences cholesterol metabolism, and ACER2 rs7022373, involved in cellular apoptosis, emerged as key genetic markers [30]. While the genotype score concept is promising, clinical translation remains distant without further validation and mechanistic exploration. Although the skeletal muscle is essential to metabolism and significantly impacts aging and chronic diseases, the genetic variations associated with the skeletal muscle remain largely unexplored [40].

Epigenome-wide association studies (EWASs) have been used for the past decade to examine deoxyribonucleic acid (DNA) methylation variations in complex diseases, making the epigenome an increasingly prominent focus of the current research [41].

The study conducted by Xu et al. (2024) examined the genetic associations with sarcopenic obesity (SO) in a large cohort from the UK Biobank using an exome-wide sequencing approach [31]. The single-variant association analysis identified 14 SNPs significantly associated with SO at the exome-wide level, all located within a single genomic locus at 1q41 [31]. The lead variant, rs1417066, was found in the LYPLAL1-AS1 gene, and ADIPOQ (adiponectin) was identified as a potential protein-level marker [31]. This study highlights both common and rare genetic variants influencing SO risk, expanding the genetic architecture of SO and suggesting novel therapeutic targets. However, this study has some limitations, including the use of a bioelectrical impedance analysis (BIA) instead of dual-energy X-ray absorptiometry (DXA), a restricted focus on White European populations, and a lack of functional validation of the identified genes [31]. The strengths of this study include its statistical power and rigorous methodology. However, critical limitations remain, such as a lack of functional validation, the failure to replicate the findings in diverse populations, and the reliance on indirect phenotyping methods. While there is clinical potential, further studies are necessary to validate the identified targets and to explore their underlying biological mechanisms.

The study conducted by Ran et al. [32] (2020) explored the genetic associations with sarcopenia in a Han Chinese population using a combined whole-exome sequencing (WES) and genome-wide association study (GWAS) approach. Among its findings, SOAT2, an enzyme in the acyl coenzyme A:cholesterol acyltransferase family, was identified as potentially exerting pleiotropic effects on body mass development [32]. Their statistical analyses revealed that SOAT2 variants, including rs2272303, rs11170413, and rs2272302, may play roles in sarcopenia risk [32]. SOAT2 has been implicated in cholesterol metabolism and obesity [32]. Although this study identified novel genetic markers for sarcopenia, certain limitations should be considered, including the relatively small discovery sample size and the variability introduced by the differences in the body composition measurement methods across the two cohorts [32]. Future work should prioritize larger discovery cohorts, direct experimental validation, and integration of functional sarcopenia measures to advance clinical relevance.

#### 4.1.4. Insulin Signaling and Glucose Metabolism

In humans, resistin is primarily expressed in the monocytes and macrophages, where it contributes to the development of insulin resistance [25]. Specific SNP haplotypes (the G-A haplotype at SNP-420 rs1862513 and SNP-358 rs3219175) may contribute to the development of latent sarcopenic obesity [25]. The definition of latent sarcopenic obesity requires further validation. Future studies should focus on standardizing these criteria, as presented in the study conducted by Ikeda et al. in 2023, and validating them in larger, more diverse populations, extending beyond the Japanese cohort, to ensure their clinical relevance and applicability [25].

#### 4.1.5. Oxidative Stress and Inflammation

Khanal et al. (2021) highlighted the genetic susceptibility to sarcopenia in obese women, with MTHFR polymorphisms potentially playing a role in muscle function and integrity [23]. Individuals with the MTHFR rs1801131 G allele exhibited a 1.9 times greater risk of sarcopenia compared to the risk in those with TT homozygotes among obese elderly individuals [23]. This association indicates that genetic variability in the MTHFR gene may influence muscle metabolism and increase susceptibility to muscle mass loss [23]. Furthermore, MTHFR rs1537516 A-allele carriers had a 2.8 times higher likelihood of developing sarcopenia compared to that in those with GG homozygotes in obese elderly women [23]. The distribution of the participants across the groups was uneven, and the decision to restrict the investigation to female participants, along with the use of a bioelectrical impedance analysis (BIA) as the body composition assessment method, represented an additional constraint [23].

Urzi et al. found that the MTHFR rs1801131 and NRF2 rs12594956 polymorphisms were significantly associated with sarcopenia risk [1]. The C allele of MTHFR was linked to a 3.3-fold increased risk, while the X allele of ACTN3 and the C allele of NRF2 also showed significant associations with a decreased muscle mass and strength [1]. Despite its significant findings, this study presents limitations, including a small sample size and a homogeneous Slovenian population, which may limit the generalizability of the results. The authors highlight the need for larger, multi-ethnic studies to validate their findings and explore the genetic contribution to sarcopenia susceptibility further [1].

While these findings highlight potential genetic markers (MTHFR rs1537516, MTHFR rs1801131, NRF2 rs12594956) related to oxidative stress and inflammation, their clinical translation requires further validation through functional studies to clarify the underlying biological pathways and through clinical trials to assess their predictive utility for risk stratification.

Khanal et al. (2020) reported that using the %SMM definition of sarcopenia, the NOS3 rs1799983 variant was identified as another influential factor, as individuals with the GG genotype faced a 2.26 times greater likelihood of developing sarcopenia compared to that for T-allele carriers, pointing to a possible link between nitric oxide signaling and muscle preservation [22]. Notably, the cohort consisted exclusively of Caucasian female participants. The observed effect sizes were modest and accompanied by wide confidence intervals. Although this SNP suggests potential for genetic risk stratification, its clinical applicability requires replication in more diverse populations.

The study conducted by Shrestha et al. in 2024 explored the association between BDKRB2 polymorphisms and physical performance and muscle mass in older adults with sarcopenia and found that in men, the rs1799722 TT genotype was associated with longer 6MWDs and a greater leg muscle mass, while the rs5810761 -9-9 genotype was associated with a lower arm fat mass [29]. Additionally, the +9+9 genotype of rs5810761 was linked to an improvement in SPPB scores at 12 months. Despite these findings, this study faced limitations, including a relatively small sample size and the absence of a control group without sarcopenia [29]. No associations were observed in women. These limitations and the absence of mechanistic data reduce the generalizability of its results. Larger and more diverse studies are needed to confirm these findings and explore their clinical application further.

#### 4.1.6. Hormonal Regulation

Khanal et al. (2020) reported that using the %SMM definition, the ESR1 rs4870044 polymorphism was strongly associated with sarcopenia, with individuals carrying the T allele exhibiting a 2.54 times higher risk compared to that in those with the CC genotype [22]. This finding highlights the potential role of the estrogen receptor pathways in muscle health, but this has still not been fully defined [22].

In contrast, Khanal et al. [22] (2020) reported that when using the SMI definition, only one genetic variant was significantly associated with sarcopenia. The TRHR rs7832552 SNP was found to play a notable role, with individuals carrying the C allele exhibiting a 2.6 times higher risk of sarcopenia than the risk in those with the TT genotype [22]. This suggests the potential involvement of the thyrotropin-releasing hormone receptor in muscle function and maintenance [22]. When sarcopenia was defined based on the EWGSOP criteria, no significant genetic associations were identified [22]. This was likely due to the low prevalence (1.3%) of sarcopenia under this definition, which limited the statistical power to detect meaningful correlations [22]. While genetic insights hold promise for personalized risk assessments, certain methodological limitations, such as the reliance on bioelectrical impedance rather than dual-energy X-ray absorptiometry or Magnetic Resonance Imaging, as well as this study’s specific focus on older women, necessitate cautious interpretation [22].

#### 4.1.7. The Cell Cycle and Regeneration

The study conducted by Shu Ran et al. [32] (2020) explored the genetic associations with sarcopenia in a Han Chinese population using a combined whole-exome sequencing (WES) and genome-wide association study (GWAS) approach. Their statistical analyses identified that the FZR1 rs740681 SNP is significantly associated with sarcopenia risk [32]. Functional annotation highlighted the role of FZR1 in regulating the cell cycle and muscle differentiation [32]. While FZR1 rs740681 represents a novel sarcopenia-associated variant, its evidence base is weakened by methodological constraints and indirect functional validation. Further replication in diverse cohorts and deeper mechanistic studies are needed to confirm its role. Its clinical application remains early-stage but deserves investigation within the scope of targeted functional genomics.

In this review, SNPs were identified in genes associated with key functional pathways such as muscle structure, function and atrophy, neurotransmission, lipid metabolism and adipogenesis, insulin signaling and glucose metabolism, oxidative stress and inflammation, hormonal regulation, and cell cycle control and tissue regeneration, underscoring the multifaceted genetic architecture underlying sarcopenia and sarcopenic obesity.

Several SNPs were frequently reported in the studies included in this review, such as FTO rs9939609, ACTN3 rs1815739, and MTHFR rs1801131, which are analyzed further below.

The studies by Khanal et al. [22] and Zhang et al. [26] provide important insights into the complex role of the FTO rs9939609 polymorphism in sarcopenia, highlighting its population-specific effects and the influence of genetic and environmental factors. In European women, the AA genotype was linked to a threefold increased risk of sarcopenia [22]. In contrast, in Tibetan populations, the TT genotype was associated with an elevated risk of sarcopenia, possibly due to unique genetic adaptations to chronic hypoxia [26]. These findings underscore the importance of considering gender-specific differences, as both studies observed stronger associations in women, suggesting that hormonal changes (such as a postmenopausal decline in estrogen) and body composition factors may contribute to the accelerated muscle loss observed in this group. The variability in the genotype-risk associations also emphasizes the need for personalized approaches in the diagnosis and treatment of sarcopenia, tailored to ethnicity, genetics, and environmental contexts. Understanding this interplay is essential for advancing our knowledge of sarcopenia and improving clinical strategies for its prevention and management across diverse populations.

In Khanal et al. [23], both ACTN3 rs1815739 and MTHFR rs1801131 were associated with sarcopenic obesity, while in Urzi et al. [1], ACTN3 rs1815739 and MTHFR rs1801131 were significantly linked to sarcopenia risk, emphasizing the need for tailored interventions to address obesity-related and age-related muscle decline. The contrasting allelic associations of ACTN3 rs1815739 and MTHFR rs1801131 observed between obese elderly women [23] and the general elderly population [1] underscore the multifactorial nature of sarcopenia and sarcopenic obesity, highlighting the necessity of context-specific genetic profiling.

Despite the recurrent identification of SNPs such as FTO rs9939609, ACTN3 rs1815739, and MTHFR rs1801131 across various studies, the existing evidence regarding their role in sarcopenia and sarcopenic obesity remains inconsistent but may suggest that these genetic variations exert biological effects across diverse populations and study designs. This recurring pattern underscores the need for further investigation into the underlying mechanisms and an evaluation of their applicability across different populations. Moreover, it highlights the importance of standardizing the study methodologies to improve the precision and comparability of the findings in the field of genetic associations related to sarcopenia and sarcopenic obesity.

The pathophysiology of sarcopenia and sarcopenic obesity is highly complex and multifactorial and has become a major focus of scientific research, with ongoing efforts to translate new insights into practice.

### 4.2. The Identification of Gaps in the Current Knowledge and Future Research Directions

This study highlights high-risk genetic profiles that could be instrumental in developing preventive strategies for sarcopenia and sarcopenic obesity. It supports the advancement of genetic screening tools for early detection and encourages further research to explore how single-nucleotide polymorphisms (SNPs) influence the progression of sarcopenia or sarcopenic obesity over time.

### 4.3. The Strengths of This Study

By ensuring a transparent and reproducible methodology for the study selection and data extraction, using PRISMA, this study minimizes bias and enhances reliability [42]. It systematically searches six databases, reducing the publication bias and increasing the comprehensiveness of the findings. This study includes the incorporation of robust methodologies such as cross-sectional studies, exome-wide association studies (EWASs), whole-exome sequencing (WES), and genome-wide association studies (GWASs). Furthermore, the inclusion of studies from diverse ethnic and geographical backgrounds provides valuable insights into the genetic variations across populations, enhancing the applicability of the results.

### 4.4. Limitations of This Study

Definitions of sarcopenia and sarcopenic obesity vary across studies employing different diagnostic criteria, which can introduce inconsistencies. The genotyping methods have varying levels of sensitivity and specificity, which may result in discrepancies in detecting SNPs. The studies included utilized diverse genotyping techniques, leading to potential variability in their results [43]. This variability makes it difficult to compare the findings across studies or replicate their results. Heterogeneity in the study populations, including differences in age and sex distribution, as well as variability in the study designs and outcome measures, may have further contributed to inconsistent findings and limited comparability. Furthermore, some genetic studies on sarcopenia and sarcopenic obesity have relatively small sample sizes, limiting their statistical power and ability to detect significant associations. Genetic associations may also differ across populations, affecting the generalizability of the findings. Another limitation of this study is that non-English databases were not included [44], which may have led to the omission of relevant data. Additionally, the studies included in our systematic review varied in sample size, and there was a potential risk of a “small study” effect, where smaller studies tend to report larger and more favorable effect sizes compared to studies with larger sample sizes [45]. Due to the clinical and methodological heterogeneity, a meta-analysis was not conducted.

## 5. Conclusions

The existing evidence on variations in SNPs in the context of sarcopenia and sarcopenic obesity lacks sufficient consistency, making study comparisons challenging. Despite inconsistencies in the published findings, genetic insights remain valuable for advancing the prevention and treatment strategies for sarcopenia and sarcopenic obesity. Understanding their genetic predictors could enhance early detection, refine risk models, and facilitate personalized interventions. To strengthen future research, studies should focus on larger, multi-ethnic cohorts; adopt longitudinal designs; adopt the same diagnostic criteria; and incorporate functional validation of SNPs.

## Figures and Tables

**Table 1:** Number of articles retrieved from databases for the search phrase “(sarcopenia OR sarcopenic obesity) AND (single nucleotide polymorphisms OR SNPs OR genetic variants)” according to the initial search filters.

Databases	Database Search Filters	Number of Results
PubMed, National Institutes of Health (NIH)	Publication years: 2015-present Language: English Species: humans Article types: all	122 results
Web of Science	Publication years: 2015-present Language: English Article types: all	137 results
Cochrane Library	Publication years: 2015-present Language: English	7 results
Scopus	Publication years: 2015-present Language: English Species: humans Article types: articles, reviews	512 results
ScienceDirect	Publication years: 2015-present Language: English Article types: research articles, case reports, reviews, mini-reviews	816 results
SpringerLink Journals	Publication years: 2015-present Language: English Article types: articles, review articles, research articles	484 results
TOTAL		2078 results

**Table 2:** Summary of included studies.

Author’s Name and Year of Publication	Country	Pathology Studied	Study Design	Sample Size	Number of SNPs Identified	References
Khanal et al. [22] 2020	United Kingdom	Sarcopenia	Cross-sectional study	307 Caucasian female patients	4 SNPs	92
Khanal et al. [23] 2021	United Kingdom	Sarcopenia Sarcopenic Obesity and Obesity	Cross-sectional study	307 Caucasian female patients	3 SNPs	97
Agostini et al. [2] 2021	Italy	Sarcopenia	Cross-sectional study	358 Caucasian patients (177 sarcopenic patients and 181 controls)	1 SNP	55
Montazeri-Najafabady et al. [24] 2022	Iran	Sarcopenia	Cross-sectional study	254 patients (65 sarcopenic patients and 189 controls)	2 SNPs	45
Ikeda et al. [25] 2023	Japan	Latent Sarcopenic Obesity	Cross-sectional study	567 participants	2 SNPs	36
Zhang et al. [26] 2021	China	Sarcopenia	Cross-sectional study	160 participants (all sarcopenic)	4 SNPs	50
Urzi et al. [1] 2021	Slovenia	Sarcopenia	Cross-sectional study	190 participants (sarcopenic group: 45 participants; control group: 145 participants)	3 SNPs	75
Bashir et al. [27] 2023	United Kingdom	Sarcopenia	Post hoc sub-study of a randomized controlled trial [28]	110 Caucasian patients (all sarcopenic)	2 SNPs	47
Shrestha et al. [29] 2024	United Kingdom	Sarcopenia	Post hoc sub-study of a randomized controlled trial [28]	136 Caucasian patients (all sarcopenic)	2 SNPs	31
Wu et al. [30] 2021	China	Sarcopenia	Genome-wide association study (GWAS)	96 participants	8 SNPs were strongly associated	52
Xu et al. [31] 2024	China	Sarcopenic Obesity	Exome-wide association study (EWAS)	282,164 participants (6890 with sarcopenic obesity, 275,274 controls)	14 SNPs	50
Ran et al. [32] 2020	China	Sarcopenia	Whole-exome sequencing (WES) and genome-wide association study (GWAS)	101 (Chinese Han), Replication: 217,822 (UK Biobank)	4 significant SNPs	39

**Table 3:** Q-Genie quality assessment of the included studies.

Studies	Items											Total Score	Quality Classification
	1	2	3	4	5	6	7	8	9	10	11		
Khanal et al. [22]	6	5	5	6	7	4	4	6	5	4	5	57/77	Good
Khanal et al. [23]	6	6	6	7	5	4	4	6	5	4	6	59/77	Good
Agostini et al. [2]	7	6	6	7	6	5	6	7	6	6	7	69/77	Good
Montazeri-Najafabady et al. [24]	6	5	5	6	4	5	4	5	4	4	6	54/77	Good
Ikeda et al. [25]	6	6	6	7	4	5	5	6	7	5	6	63/77	Good
Zhang et al. [26]	6	6	6	7	4	4	4	5	5	5	6	58/77	Good
Urzi et al. [1]	6	6	5	6	5	6	5	6	5	5	6	61/77	Good
Bashir et al. [27]	7	6	5	6	5	5	4	5	6	6	6	61/77	Good
Shrestha et al. [29]	6	7	4	5	4	5	4	4	4	5	5	53/77	Good
Wu et al. [30]	7	6	5	7	7	6	3	5	7	5	7	65/77	Good
Xu et al. [31]	7	6	6	6	7	6	7	6	7	6	7	71/77	Good
Ran et al. [32]	7	6	7	7	6	6	7	6	7	6	7	72/77	Good

Items: 1: Rationale for the study; 2: Selection and definition of outcome of interest; 3: Selection and comparability of comparison groups; 4: Technical classification of the exposure; 5: Non-technical classification of the exposure; 6: Other sources of bias; 7: Sample size and power; 8: A priori planning of analysis; 9: Statistical methods and control for confounding; 10: Testing of assumptions and inferences for genetic analyses; 11: Appropriateness of inferences drawn from results [33]. Scoring of 1 to 7, with 1 being poor, 3 being good, 5 being very good, and 7 being excellent. For studies with control groups, scores ≤ 35 indicate poor-quality studies, >35 and ≤45 indicate studies of moderate quality, and >45 indicate good-quality studies. For studies without control groups, scores ≤ 32 indicate poor-quality studies, >32 and ≤40 indicate studies of moderate quality, and >40 indicate good-quality studies [20].

**Table 4:** SNPs identified.

Author’s Name	Pathology Studied	SNPs Identified	Mentions
Khanal et al. [22]	Sarcopenia	FTO rs9939609, ESR1 rs4870044, NOS3 rs1799983, TRHR rs7832552	-SNPs identified vary depending on the diagnostic criteria for sarcopenia; -Exclusive focus on female participants.
Khanal et al. [23]	Sarcopenia Sarcopenic obesity Obesity	ACTN3rs1815739, MTHFR rs1801131, MTHFR rs1537516	-SNPs identified were only significantly associated with sarcopenic obesity; -Groups based on sarcopenia and obesity status were not equally distributed; -Exclusive focus on female participants.
Agostini et al. [2]	Sarcopenia	SNAP-25 rs363050	-The SNP identified is associated with sarcopenia, but the assessment was based on physical performance and muscle strength, while muscle mass was not evaluated using imaging methods, which are considered the gold standard.
Montazeri-Najafabady et al. [24]	Sarcopenia	TP53 exon 4 Arg72pro rs1042522, Intron 3 16-bp Del/Ins rs17878362	-TP53 codon 72 influences the risk of sarcopenia; -rs17878362 (16-bp Del/Ins) is technically a structural variation (Indel) rather than a traditional SNP, but it is considered a genetic polymorphism that affects gene function and has not been directly associated with sarcopenia.
Ikeda et al. [25]	Latent sarcopenic obesity	SNP-420 rs1862513, SNP-358 rs3219175	-SNPs identified are associated with an increased risk of latent sarcopenic obesity; -The definition of latent sarcopenic obesity requires further validation.
Zhang et al. [26]	Sarcopenia	FTO rs9939609, FTO rs9936385, ACVR2B rs2276541, IRS1 rs294365	-FTO rs9939609 and rs9936385 were significantly associated with lower limb skeletal muscle mass and sarcopenia, particularly in Tibetan women; -ACVR2B rs2276541 and IRS1 rs2943656 showed no significant association with sarcopenia.
Urzi et al. [1]	Sarcopenia	MTHFR rs1801131, ACTN3 rs1815739, NRF2 rs12594956	-The SNPs identified were significantly associated with sarcopenia risk.
Bashir et al. [27]	Sarcopenia	ACVR1B rs10783486, ACVR1B rs2854464	-The SNPs were linked to height and limb fat mass, rather than muscle mass or strength, in older men with sarcopenia.
Shrestha et al. [29]	Sarcopenia	BDKRB2 rs1799722, BDKRB2 rs5810761	-BDKRB2 polymorphisms are associated with physical performance and muscle mass.
Wu et al. [30]	Sarcopenia	OSBPL3 rs10282247, ACER2 rs7022373, GM2A rs60274968, TMEM14A rs6905523, IZUMO3 rs57247929, OR5D18 rs11231180, OR4A9P rs118154365, ATXN8OS rs9572533	-Notably, OSBPL3 rs10282247 (influencing cholesterol metabolism) and ACER2 rs7022373 (involved in cellular apoptosis) were identified as key genetic markers.
Xu et al. [31]	Sarcopenic obesity	LYPLAL1-AS1 rs1417066, LYPLAL1-AS1 rs11205303, LYPLAL1-AS1 rs12138590, LYPLAL1-AS1 rs13374518, LYPLAL1-AS1 rs147871, LYPLAL1-AS1 rs188491278, LYPLAL1-AS1 rs34360, LYPLAL1-AS1 rs34594323, LYPLAL1-AS1 rs35706747, LYPLAL1-AS1 rs3736533, LYPLAL1-AS1 rs4980745, LYPLAL1-AS1 rs55687493, LYPLAL1-AS1 rs6670062, LYPLAL1-AS1 rs76293177	-The lead variant, rs1417066, in the LYPLAL1-AS1 gene was the most significant genetic marker for SO.
Ran et al. [32]	Sarcopenia	FZR1 rs740681, SOAT2 rs2272303, SOAT2 rs11170413, SOAT2 rs2272302	-Additionally, FZR1 plays a key role in cell cycle regulation and muscle differentiation, while SOAT2 is involved in cholesterol metabolism and obesity.

FTO (Fat mass and obesity-associated gene). ESR1 (estrogen receptor 1). NOS3 (Endothelial nitric oxide synthase 1). TRHR (thyrotropin-releasing hormone receptor). ACTN3 (alpha actinin cardiac muscle 3). MTHFR (Methylenetetrahydrofolate reductase). SNAP-25 (synaptosomal-associated protein of 25 kDa). TP53 (Tumor suppressor protein 53). Del/Ins (Deletion/Insertion). CVR2B (activin receptor type-2B). IRS1 (Insulin receptor substrate 1). NRF2 (Nuclear factor erythroid 2-related factor 2). ACVR1B (activin receptor type-1B). BDKRB2 (Bradykinin Receptor B2). SO (sarcopenic obesity). OSBPL3 (Oxysterol binding protein-like). ACER2 (Alkaline ceramidase 2). GM2A (Ganglioside activator). TMEM14A (Transmembrane Protein 14A). IZUMO3 (IZUMO family member 3). OR5D18 (Olfactory receptor family 5 subfamily D member 18). OR4A9P (Olfactory receptor, family 4, subfamily A, member 9 pseudogene). ATXN8OS (Ataxin-8 opposite strand). LYPLAL1-AS1 (Lysophospholipase Like 1-Antisense RNA 1). FZR1 (Fizzy-related protein homolog). SOAT2 (Sterol O-acyltransferase 2).

## Supplementary Materials

The following supporting information can be downloaded at https://www.mdpi.com/article/10.3390/medicina61050866/s1, File S1: Detailed scoring for tools of risk assessment; File S2: Functional pathways distribution of the relevant SNPs.

## List of Abbreviations

The following abbreviations are used in this manuscript (in alphabetical order):

ACE	Angiotensin-Converting Enzyme
ACER2	Alkaline ceramidase 2
ACTN3	Alpha actinin cardiac muscle 3
ACVR1B	Activin receptor type-1B
ACVR2B	Activin receptor type-2B
ADIPOQ	Adiponectin
ALM/W	Appendicular lean mass adjusted for weight
ATXN8OS	Ataxin-8 opposite strand
AWGS	Asian Working Group for Sarcopenia
BDKRB2	Bradykinin Receptor B2
BIA	Bioelectrical impedance analysis
Del/Ins	Deletion/Insertion
DNA	Deoxyribonucleic acid
DXA	Dual-energy X-ray absorptiometry
EASO	European Association for the Study of Obesity
ESPEN	European Society for Clinical Nutrition and Metabolism
ESR1	Estrogen receptor 1
EWAS	Epigenome-wide association study
EWGSOP	European Working Group on Sarcopenia in Older People
FM%	Fat mass percentage
FTO	Fat mass and obesity-associated gene
FZR1	Fizzy-related protein homolog
GM2A	Ganglioside activator
GS	Gait speed
GWAS	Genome-wide association study
HGS	Handgrip strength
IRS1	Insulin receptor substrate 1
IZUMO3	IZUMO family member 3
KASP	Kompetitive Allele-Specific PCR
LYPLAL1-AS1	Lysophospholipase Like 1-Antisense RNA 1
MiRNA	Microribonucleic acid
MTHFR	Methylenetetrahydrofolate reductase
6MWD	Six-Minute Walk Distance
NIH	National Institutes of Health
NOS3	Endothelial nitric oxide synthase 1
NRF2	Nuclear factor erythroid 2-related factor 2
OR4A9P	Olfactory receptor, family 4, subfamily A, member 9 pseudogene
OR5D18	Olfactory receptor family 5 subfamily D member 18
OSBPL3	Oxysterol binding protein-like
PCR	Polymerase chain reaction
PRISMA	Preferred Reporting Items for Systematic Reviews and Meta-Analyses
qPCR	Quantitative PCR
RFLP	Restriction fragment length polymorphism
SGS	Sarcopenia genetic risk score
SMI	Skeletal muscle mass index
%SMM	%Skeletal muscle mass
SNAP-25	Synaptosomal-associated protein of 25 kDa
SNPs	Single-nucleotide polymorphisms
SO	Sarcopenic obesity
SOAT2	Sterol O-acyltransferase
2SPPB	Short Physical Performance Battery
TMEM14A	Transmembrane Protein 14A
TP53	Tumor suppressor protein 53
TRHR	Thyrotropin-releasing hormone receptor
VFA	Visceral fat area
WES	Whole-exome sequencing
WLBM	Whole lean body mass

## References

[1] Urzi F., Pokorny B., Buzan E. (2021). Pilot Study on Genetic Associations with Age-Related Sarcopenia. Front. Genet..

[2] Agostini S., Mancuso R., Costa A.S., Guerini F.R., Trecate F., Miglioli R., Menna E., Arosio B., Clerici M. (2021). Sarcopenia associates with SNAP-25 SNPs and a miRNAs profile which is modulated by structured rehabilitation treatment. J. Transl. Med..

[3] Villa O., Stuhr N.L., Yen C.-A., Crimmins E.M., Arpawong T.E., Curran S.P. (2022). Genetic variation in ALDH4A1 is associated with muscle health over the lifespan and across species. eLife.

[4] Cruz-Jentoft A.J., Sayer A.A. (2019). Sarcopenia. Lancet.

[5] Walston J.D. (2012). Sarcopenia in older adults. Curr. Opin. Rheumatol..

[6] Stanciu L.-E., Iliescu M.-G., Oprea C., Ionescu E.-V., Petcu A., Ciortea V.M., Petcu L.C., Apostol S., Nedelcu A.-D., Motoașcă I. (2023). The Impact of Complex Rehabilitation Treatment on Sarcopenia—Pathology with an Endocrine Morphological Substrate and Musculoskeletal Implications. Medicina.

[7] Wei S., Nguyen T.T., Zhang Y., Ryu D., Gariani K. (2023). Sarcopenic obesity: Epidemiology, pathophysiology, cardiovascular disease, mortality, and management. Front. Endocrinol..

[8] Donini L.M., Busetto L., Bischoff S.C., Cederholm T., Ballesteros-Pomar M.D., Batsis J.A., Bauer J.M., Boirie Y., Cruz-Jentoft A.J., Dicker D. (2022). Definition and Diagnostic Criteria for Sarcopenic Obesity: ESPEN and EASO Consensus Statement. Obes. Facts.

[9] Prado C.M., Batsis J.A., Donini L.M., Gonzalez M.C., Siervo M. (2024). Sarcopenic obesity in older adults: A clinical overview. Nat. Rev. Endocrinol..

[10] Kalinkovich A., Livshits G. (2017). Sarcopenic obesity or obese sarcopenia: A cross talk between age-associated adipose tissue and skeletal muscle inflammation as a main mechanism of the pathogenesis. Ageing Res. Rev..

[11] Batsis J.A., Villareal D.T. (2018). Sarcopenic obesity in older adults: Aetiology, epidemiology and treatment strategies. Nat. Rev. Endocrinol..

[12] Aslam M.A., Ma E.B., Huh J.Y. (2023). Pathophysiology of sarcopenia: Genetic factors and their interplay with environmental factors. Metabolism.

[13] da Silva J.R.D., Freire I.V., Ribeiro Í.J., Dos Santos C.S., Casotti C.A., Dos Santos D.B., Barbosa A.A.L., Pereira R. (2018). Improving the comprehension of sarcopenic state determinants: An multivariate approach involving hormonal, nutritional, lifestyle and genetic variables. Mech. Ageing Dev..

[14] Semenova E.A., Pranckevičienė E., Bondareva E.A., Gabdrakhmanova L.J., Ahmetov I.I. (2023). Identification and Characterization of Genomic Predictors of Sarcopenia and Sarcopenic Obesity Using UK Biobank Data. Nutrients.

[15] Wilkinson D., Piasecki M., Atherton P. (2018). The age-related loss of skeletal muscle mass and function: Measurement and physiology of muscle fibre atrophy and muscle fibre loss in humans. Ageing Res. Rev..

[16] Carmelli D., Reed T. (2000). Stability and change in genetic and environmental influences on hand-grip strength in older male twins. J. Appl. Physiol..

[17] Jin H., Yoo H.J., Kim Y.A., Lee J.H., Lee Y., Kwon S.-H., Seo Y.J., Lee S.H., Koh J.-M., Ji Y. (2022). Unveiling genetic variants for age-related sarcopenia by conducting a genome-wide association study on Korean cohorts. Sci. Rep..

[18] Bilski J., Pierzchalski P., Szczepanik M., Bonior J., Zoladz J.A. (2022). Multifactorial Mechanism of Sarcopenia and Sarcopenic Obesity. Role of Physical Exercise, Microbiota and Myokines. Cells.

[19] Page M.J., McKenzie J.E., Bossuyt P.M., Boutron I., Hoffmann T.C., Mulrow C.D., Shamseer L., Tetzlaff J.M., Akl E.A., Brennan S.E. (2021). The PRISMA 2020 statement: An updated guideline for reporting systematic reviews. BMJ.

[20] Sohani Z.N., Meyre D., de Souza R.J., Joseph P.G., Gandhi M., Dennis B.B., Norman G., Anand S.S. (2015). Assessing the quality of published genetic association studies in meta-analyses: The quality of genetic studies (Q-Genie) tool. BMC Genet..

[21] Sohani Z.N., Sarma S., Alyass A., de Souza R.J., Robiou-du-Pont S., Li A., Mayhew A., Yazdi F., Reddon H., Lamri A. (2016). Empirical evaluation of the Q-Genie tool: A protocol for assessment of effectiveness. BMJ Open.

[22] Khanal P., He L., Stebbings G., Onambele-Pearson G.L., Degens H., Williams A., Thomis M., Morse C.I. (2020). Prevalence and association of single nucleotide polymorphisms with sarcopenia in older women depends on definition. Sci. Rep..

[23] Khanal P., Williams A., He L., Stebbings G., Onambele-Pearson G., Thomis M., Degens H., Morse C. (2021). Sarcopenia, Obesity, and Sarcopenic Obesity: Relationship with Skeletal Muscle Phenotypes and Single Nucleotide Polymorphisms. J. Clin. Med..

[24] Montazeri-Najafabady N., Dabbaghmanesh M.H., Nasimi N., Sohrabi Z., Estedlal A., Asmarian N. (2022). Importance of TP53 codon 72 and intron 3 duplication 16 bp polymorphisms and their haplotypes in susceptibility to sarcopenia in Iranian older adults. BMC Geriatr..

[25] Ikeda Y., Kawamura R., Takata Y., Tabara Y., Maruyama K., Takakado M., Hadate T., Ohashi J., Saito I., Ogawa Y. (2023). Resistin G-A haplotype at SNP-420/-358 is associated with the latent sarcopenic obesity index in the toon genome study. J. Diabetes Investig..

[26] Zhang X., Ye L., Li X., Chen Y., Jiang Y., Li W., Wen Y. (2021). The association between sarcopenia susceptibility and polymorphisms of FTO, ACVR2B, and IRS1 in Tibetans. Mol. Genet. Genom. Med..

[27] Bashir T., Achison M., Adamson S., Akpan A., Aspray T., Avenell A., Band M.M., Burton L.A., Cvoro V., Donnan P.T. (2023). Activin type I receptor polymorphisms and body composition in older individuals with sarcopenia-Analyses from the LACE randomised controlled trial. PLoS ONE.

[28] Achison M., Adamson S., Akpan A., Aspray T., Avenell A., Band M.M., Bashir T., Burton L.A., Cvoro V., LACE Study Group (2022). Effect of perindopril or leucine on physical performance in older people with sarcopenia: The LACE randomized controlled trial. J. Cachex-Sarcopenia Muscle.

[29] Shrestha A., Bashir T., Achison M., Adamson S., Akpan A., Aspray T., Avenell A., Band M.M., Burton L.A., Cvoro V. (2024). Association of bradykinin receptor 2 (BDKRB2) variants with physical performance and muscle mass: Findings from the LACE sarcopenia trial. PLoS ONE.

[30] Wu S., Chen W. (2021). A Genome-Wide Association Study Identifies Novel Risk Loci for Sarcopenia in a Taiwanese Population. J. Inflamm. Res..

[31] Xu Q., Zhao Q.-G., Ma X.-L., Yan S.-S., Han B.-X., Song Z.-T., Bu F., Li K., Zhang L., Pei Y.-F. (2024). Exome-Wide Sequencing Study Identified Genetic Variants Associated With Sarcopenic Obesity. J. Gerontol. A Biol. Sci. Med. Sci..

[32] Ran S., He X., Jiang Z.-X., Liu Y., Zhang Y.-X., Zhang L., Gu G.-S., Pei Y., Liu B.-L., Tian Q. (2020). Whole-exome sequencing and genome-wide association studies identify novel sarcopenia risk genes in Han Chinese. Mol. Genet. Genom. Med..

[33] Pratt J., Boreham C., Ennis S., Ryan A.W., De Vito G. (2019). Genetic Associations with Aging Muscle: A Systematic Review. Cells.

[34] Higgins J.P., Morgan R.L., Rooney A.A., Taylor K.W., Thayer K.A., Silva R.A., Lemeris C., Akl E.A., Bateson T.F., Berkman N.D. (2024). A tool to assess risk of bias in non-randomized follow-up studies of exposure effects (ROBINS-E). Environ. Int..

[35] Beaudart C., McCloskey E., Bruyère O., Cesari M., Rolland Y., Rizzoli R., Araujo de Carvalho I., Amuthavalli Thiyagarajan J., Bautmans I., Bertière M.C. (2016). Sarcopenia in daily practice: Assessment and man-agement. BMC Geriatr..

[36] Cawthon P.M., Visser M., Arai H., Ávila-Funes J.A., Barazzoni R., Bhasin S., Binder E., Bruyère O., Cederholm T., Chen L.-K. (2022). Defining terms commonly used in sarcopenia research: A glossary proposed by the Global Leadership in Sarcopenia (GLIS) Steering Committee. Eur. Geriatr. Med..

[37] Brinkmalm A., Brinkmalm G., Honer W.G., Frölich L., Hausner L., Minthon L., Hansson O., Wallin A., Zetterberg H., Blennow K. (2014). SNAP-25 is a promising novel cerebrospinal fluid biomarker for synapse degeneration in Alzheimer’s disease. Mol. Neurodegener..

[38] Al-Daghri N.M., Costa A.S., Alokail M.S., Zanzottera M., Alenad A.M., Mohammed A.K., Clerici M., Guerini F.R. (2016). Synaptosomal Protein of 25 kDa (*Snap25*) Polymorphisms Associated with Glycemic Parameters in Type 2 Diabetes Patients. J. Diabetes Res..

[39] Tam V., Patel N., Turcotte M., Bossé Y., Paré G., Meyre D. (2019). Benefits and limitations of genome-wide association studies. Nat. Rev. Genet..

[40] Yoon K.J., Yi Y., Do J.G., Kim H.-L., Lee Y.-T., Kim H.-N. (2021). Variants in NEB and RIF1 genes on chr2q23 are associated with skeletal muscle index in Koreans: Genome-wide association study. Sci. Rep..

[41] Wei S., Tao J., Xu J., Chen X., Wang Z., Zhang N., Zuo L., Jia Z., Chen H., Sun H. (2021). Ten Years of EWAS. Adv. Sci..

[42] Iliescu M.G., Stanciu L.-E., Uzun A.-B., Cristea A.-E., Motoască I., Irsay L., Iliescu D.M., Vari T., Ciubean A.D., Caraban B.M. (2024). Assessment of Integrative Therapeutic Methods for Improving the Quality of Life and Functioning in Cancer Patients—A Systematic Review. J. Clin. Med..

[43] Nasui B.A., Talaba P., Nasui G.A., Sirbu D.M., Borda I.M., Pop A.L., Ciortea V.M., Irsay L., Purcar-Popescu A.I., Cinteza D. (2022). The Influence of Diet and Physical Activity on Oxidative Stress in Romanian Females with Osteoarthritis. Nutrients.

[44] Pan J.T., See K.C. (2024). Criteria for stopping high-flow nasal oxygen for acute hypoxemic respiratory failure: A systematic review of randomized controlled trials. Anesthesiol. Perioper. Sci..

[45] Xu Y., Han Y., Zhuang H., Fei F., Zheng T., Yu H. (2024). Effect of ultrasound-guided recruitment maneuver on atelectasis: A systematic review and meta-analysis of randomized controlled trials. Anesthesiol. Perioper. Sci..

