# Supplementary material for: Genetic Patterns Related with the Development and Progression of Sarcopenia and Sarcopenic Obesity: A Systematic Review
Source: Medicina (Kaunas). 2025 May 8;61(5):866. doi: 10.3390/medicina61050866 (PMC12113501; doi:10.3390/medicina61050866)
Supplement: Supplementary file 1 [file medicina-61-00866-s001.zip › medicina-3599384- File S1.pdf]

Detailed scoring for tools of risk assessment

Table S1- Justification of Final Q-Genie Item Scoring

Studies	***Justification of Items***										
	**1**	**2**	**3**	**4**	**5**	**6**	**7**	**8**	**9**	**10**	**11**
Khanal et al. [22]	*6/7 Strong rationale by emphasizing the variability in sarcopenia prevalence based on definitions and the need to explore genetic associations. A minor deduction arises from the absence of a genome-wide approach (GWAS) or broader genetic*	*5/7 EWGSOP criteria and age adjustments are well-defined. However, the use of BIA (less precise than DXA) and lack of evaluator blinding during genotyping slightly reduce the score.*	*5/7 Groups are demographically balanced, but insufficient details on controlling ethnic or environmental factors introduce potential bias.*	*6/7 Robust genotyping methods (TaqMan) with Hardy-Weinberg Equilibrium verification. Minor deductions for incomplete reporting of call rates or minor errors.*	*7/7 Centralized and standardized genotyping procedures with no evidence of bias warrant full marks.*	*4/7 Acknowledges limitations (e.g., BIA use, sample size) but lacks thorough discussion of confounders (e.g., diet, physical activity).*	*4/7 The sample of participants is adequate but insufficient for complex genetic analyses. No a priori power calculation further limits the score.*	*6/7 Clear statistical planning (logistic regression, age adjustments) but lacks sensitivity analyses or external validation.*	*5/7 Adjustments for age and sex are present, but partial correction for multiple testing reduces validity.*	*4/7 Is verified, but no tests for consanguinity, haplotypes, or gene-environment interactions limit depth.*	*5/7 Conclusions are cautious, highlighting the need for larger validation studies. Clinical implications are discussed superficially.*

	variant exploration.										
Khanal et al.[23]	*6/7 The study provides a clear rationale by investigating genetic associations with sarcopenia in obese elderly women. However, it uses a candidate gene approach (non-GWAS) without broader exploration of genetic variants, slightly limiting novelty.*	*6/7 Outcomes (sarcopenia/obesity/sarcopenic obesity) were defined using validated criteria (SMI, HGS, BF%), but BIA instead of DXA for muscle mass can introduces measurement bias.*	*6/7 Groups were well-defined, but differences in the number of participants between groups may limit comparability.*	*7/7 Genotyping methods (TaqMan assays) were robust, with high call rates (>99%) and Hardy-Weinberg Equilibrium verification was performed. Procedures adhered to best practices, warranting full marks.*	*5/7 Centralized genotyping ensured consistency, but missing protocols lowered the score.*	*4/7 Acknowledged limitations (e.g., small sample, BIA use) but lacked discussion of confounders (e.g., diet, physical activity). Limited exploration of selection or measurement biases.*	*4/7 The sample of participants is modest for genetic analyses. No a priori power calculation was reported, reducing confidence in detecting true associations.*	*6/7 Statistical methods (ANCOVA, logistic regression) were predefined, and covariates (age/height) were justified. Missing sensitivity analyses or external validation limited the score.*	*5/7 Adjusted for age/height but lacked corrections for multiple testing.*	*4/7 Is verified, but no tests for consanguinity, haplotype validation, or population stratification. Limited adherence to genetic analysis standards.*	*6/7 Conclusions align with results, highlighting sarcopenia-obesity genetic links. However, clinical relevance and mechanistic insights (e.g., functional SNP roles) were underexplored.*
Agostini et al. [2]	*7/7 The study provides a clear rationale*	*6/7 The diagnostic criteria for sarcopenia*	*6/7 Groups were well-defined, but recruitment*	*7/7 The genotyping method (TaqMan*	*6/7 It is not clearly specified whether*	*5/7 The study mentions confounding factors*	*6/7 The sample size of patients*	*7/7 The study follows a clear*	*6/7 The analyses are robust*	*6/7 The study does not mention*	*7/7 The conclusions are supported*

	regarding the link between SNAP-25 and sarcopenia, explaining why this SNP and the selected miRNAs were investigated.	*are based on EWGSOP standards, but no imaging is included for muscle mass measurement, which would have been ideal.*	*from a single center may introduce bias.*	*SNP Genotyping Assay) was robust, and Hardy-Weinberg Equilibrium verification was performed.*	*genotyping was conducted in randomized batches.*	*such as comorbidities but does not fully address all possible sources of bias.*	*and controls is decent; however, a larger cohort would have provided stronger analytical power.*	*analysis plan, including multiple regression models and appropriate statistical corrections.*	*(ANOVA, regression analyses), but a principal component analysis to control for potential genetic variations would have been beneficial.*	*whether potential consanguinity relationships were tested.*	*by the data, and the study suggests potential biomarkers for sarcopenia.*
Montazeri-Najafabady et al.[24]	*6/7 Solid justification based on the role of TP53 in muscle homeostasis and previous studies.*	*5/7 Sarcopenia defined according to EWGSOP. However, the use of BIA (less precise than DXA) and lack of evaluator blinding during*	*5/7 Controls are describe, but the selection procedure is not detailed.*	*6/7 Genotyping methods (PCR-RFLP) are clearly described, but Hardy-Weinberg verification was neglected.*	*4/7 No mention of sample randomization.*	*5/7 Limitations (small sample size) are acknowledged, but discussion of other bias sources is superficial.*	*4/7 Modest sample, with no a priori power calculation.*	*5/7 Statistical methods are described, but the analysis plan is not explicitly stated in*	*4/7 Adjustments for age and sex were made, but no corrections for multiple testing or other*	*4/7 No verification of Hardy-Weinberg equilibrium and no details on haplotype inference.*	*6/7 Conclusions are supported by the results, with acknowledgment of the study's limitations.*

		genotyping slightly reduce the score.						*advance.*	*confounders.*		
Ikeda et al.[25]	*6/7 Although this is not a GWAS, the rationale for the study (which investigates the association between haplotypes of the RETN gene and latent sarcopenic obesity) is well supported by existing literature.*	*6/7 The latent sarcopenic obesity index is clearly defined. The measurements were standardized (body composition analyzer, dynamometer), and the participants are from a longitudinal study (Toon Genome Study), reducing the risk of bias.*	*6/7 The comparison groups (G–A homozygotes vs. C–G homozygotes) are well-defined.*	*7/7 Genotyping was performed using robust methods (TaqMan, Pyrosequencing), with success rates >99%.*	*4/7 It is not mentioned whether genotyping was performed in a blinded manner or in randomized batches, which could introduce bias.*	*5/7 Limitations (small sample size) are acknowledged, but discussion of other bias sources is superficial.*	*5/7 The sample size of participants is adequate for a genetic study, but no a priori statistical power analysis is mentioned.*	*6/7 The analytical plan is detailed: logistic regression adjusted for confounders (age, sex, comorbidities. The results are fully reported, including in supplementary tables.*	*7/7 The statistical methods are appropriate (ANCOVA, Tukey test, logistic regression). Adjustments for confounding and corrections for multiple testing are applied.*	*5/7 Haplotypes were estimated using the EM algorithm (standard method), but the verification of consanguinity relationships or concordance between sex/ethnicity is not mentioned.*	*6/7 The conclusions are supported by the results: the association between the G–A haplotype and latent sarcopenic obesity is plausible, and the role of TNF-α is highlighted through gene expression analyses.*
Zhang et al. [26]	*6/7 The study presents a solid scientific rationale regarding*	*6/7 Sarcopenia is well-defined according to standard*	*6/7 The control group and sarcopenia group are well-defined. There is no*	*7/7 DNA was properly extracted, and PCR was used for*	*4/7 There are no mentions of sample randomi*	*4/7 The limitations are insufficiently described*	*4/7 Relatively small sample, but the study discusses*	*5/7 The methodology is clearly described, but*	*5/7 Logistic regression and chi-square*	*5/7 There is no mention of consangu*	*6/7 The conclusions are supported by data. The authors*

	the impact of hypoxia on muscle mass and the predisposition of Tibetans to sarcopenia.	*criteria (AWGS).*	*detailed information about the method of group selection.*	*genotyping.The study adheres to Hardy-Weinberg principles for genotype validation.*	*zation to avoid bias.*	*and lack depth.*	*the statistical significance of the results. No a priori statistical power calculation is mentioned.*	*no sensitivity analysis is mentioned.*	*tests are used for association. No adjustments for multiple testing are mentioned.*	*inity testing.*	*discuss the differences between this study and previous ones.*
Urzi et al. [1]	*6/7 The authors presented a clear rationale for the selection of candidate genes (MTHFR, ACTN3, NRF2, etc.), based on previous studies and biological evidence.*	*6/7 Sarcopenia was defined according to EWGSOP criteria, using standardized measurements (muscle mass, walking speed, grip strength).*	*5/7 The groups (sarcopenic vs. non-sarcopenic) were drawn from the same population (Caucasians, similar age, nursing homes). There is no mention of blinding for genotyping or detailed procedures for control selection.*	*6/7 Genotyping methods (KASP assay) are described in detail, with acceptable amplification (93.5%) and quality (98%) rates. Hardy-Weinberg equilibrium was tested (except for CX3CR1, which was excluded*	*5/7 Details on evaluator blinding for genotyping or sample randomization are missing. Genotyping was performed in batches, but the consistency of methods between batches*	*6/7 The authors acknowledge significant limitations: small sample size, lack of analysis by sex, and potential bias due to Hardy-Weinberg deviation in MTHFR in the sarcopenic group. The discussion is balanced.*	*5/7 The limited sample size reduces generalizability.*	*6/7 Analyses were planned a priori (logistic regression, ANOVA) and fully reported, including in supplementary tables. Interactions or predefined subgroups are missing.*	*5/7 They adjusted for age, sex, and physical activity, but did not apply corrections for multiple testing, increasing the risk of false-positive results.*	*5/7 Consanguinity relationships or haplotype validation were not tested. Sex and ethnicity were partially controlled.*	*6/7 The conclusions are moderated by the sample size, but the results are presented with caution. The study highlights the need for validation in larger studies.*

				from analysis). No mention of sample randomization.	*is not specified.*						
**Bashir et al. [27]**	*7/7 The study provides a clear hypothesis linking ACVR1B polymorphisms to body composition in sarcopenia, grounded in prior evidence of activin/myostatin signaling in muscle biology. Gene selection aligns with pathways implicated in muscle atrophy and fat*	*6/7 Sarcopenia was defined using EWGSOP criteria.*	*5/7 Participants were sarcopenic, limiting comparisons. Selection criteria (age ≥70, EWGSOP diagnosis) are outlined, but details on control group absence and potential selection bias (e.g., exclusion of comorbid patients) reduce the score.*	*6/7 DNA extraction (QIAamp kit) and genotyping (Taqman qPCR) methods are appropriate. Hardy-Weinberg equilibrium was tested.*	*5/7 No mention of blinded genotyping or sample randomization (e.g., case/control plate distribution). Batch processing details are absent, raising concerns about technical bias.*	*5/7 Acknowledges limitations (small sample size, cross-sectional design, collider bias) but lacks discussion of selection bias or population stratification.*	*4/7 Small sample with no a priori power calculation for genetic associations. The study is underpowered to detect moderate effects, increasing error risk.*	*5/7 Statistical methods (ANCOVA, Mann-Whitney U) are described, but no adjustment for multiple testing increases false-positive risk. Supplementary tables are used but not fully integrated.*	*6/7 Adjusted for height as a confounder and used appropriate non-parametric tests. However, missing data and unaddressed multiple comparisons lower the score.*	*6/7 The Genotype-Tissue Expression (GTEx) data validated allele expression.*	*6/7 Conclusions are cautious and align with results.Extrapolation to clinical applications is premature without replication.*

	accumulation. A GWAS approach is not used, but candidate genes are very well-rationalized.										
**Shrestha et al. [29]**	*6/7 The study provides a strong rationale by linking BDKRB2 variants to muscle performance and sarcopenia, supported by prior evidence. It does not explicitly address potential alternative hypotheses or GWAS-specific rationale, slightly*	*7/7 Outcomes were objectively defined using validated tools.*	*4/7 Lack of an external control group.*	*5/7 Genotyping methods are described, but details such as call rate or genotyping errors are missing.*	*4/7 Concerns about technical bias.*	*5/7 The study acknowledged limitations (e.g., small sample size, early trial termination) but did not fully address environmental confounders (e.g., physical activity, diet).*	*4/7 The small sample size, early trial termination, and absence of a priori power calculation significantly reduced statistical power, increasing the risk of errors.*	*4/7 Analyses were described but lacked adjustments for multiple testing, leading to potential false positives. Selective reporting of post hoc subgroup analyses further impacted this score.*	*4/7 Appropriate tests (ANOVA, Kruskal-Wallis) were used, but failure to correct for multiple comparisons and incomplete handling of missing data weakened rigor.*	*5/7 No checks were performed for population stratification.No checks for consanguinity or ethnicity/sex.*	*5/7 Conclusions were cautious and highlighted the need for replication in larger cohorts. However, overinterpretation of gender-specific findings (small male subgroup) and lack of mechanistic exploration limited this score.*

	reducing the score.										
Wu et al.[30]	*7/7 The study has a clear rationale, investigating genetic associations with sarcopenia in elderly Asian adults. It provides justification for the selection of a GWAS and clearly defines the genetic importance of sarcopenia.*	*6/7 The method used for measuring muscle mass is bioelectrical impedance (BIA), which is less accurate than dual-energy X-ray absorptiometry (DXA).*	*4/7 The comparison groups are well-defined, including healthy individuals and those with sarcopenia.The studied population is limited to Taiwanese, which reduces the generalizability of the results to other ethnicities.*	*7/7 Genotyping was performed using the Affymetrix Axiom Genome-Wide TWB 2.0 array, adapted for the Taiwanese population .Strict quality control of genotyping.*	*7/7 Genotyping was performed in large batches, reducing possible errors.*	*6/7 The study discusses potential sources of bias, including limitations in muscle mass measurement and lack of ethnic diversity.*	*3/7 The sample size is very small for a GWAS, which limits the statistical power of the study and the ability to detect rare genetic variants.*	*5/7 The study clearly planned the analysis methods and reported all tests performed.No replication was conducted in an independent dataset.*	*7/7 ppropriate statistical methods were used for the GWAS analysis, including adjustment for multiple testing and principal component analysis for genetic confounding control.*	*5/7 Hypotheses regarding the genetic contribution to sarcopenia were tested, and SNPs associated with multiple traits were identified. Epigenetic effects or functional mechanisms of the identified variants were not explored.*	*7/7 The conclusions are supported by data and indicate the need for future studies for validation.*
Xu et al.[31]	*7/7 The study presents a clear hypothesi*	*6/7 SO was defined using validated*	*6/7 Controls were rigorously defined (no*	*6/7 Genotyping and imputation protocols*	*7/7 Genotyping was performed using*	*6/7 The study acknowledges limitations.*	*7/7 The large sample size*	*6/7 Analytical methods (REGE*	*7/7 Confounders were approp*	*6/7 Key assumptions were tested,*	*7/7 Conclusions align with results.imitations are*

	s and rationale, utilizing an exome-wide approach to identify genetic variants associated with sarcopenic obesity (SO). The rationale for focusing on both common and rare variants is well-explained, and the use of UK Biobank data aligns with a hypothesis-free design typical of GWAS/exome-wide studies.	*criteria (ESPEN/EASO consensus). Additionally, the cross-sectional design and inclusion of participants aged 37–73 (vs. older adults) may limit generalizability.*	*obesity/sarcopenia at any visit), and propensity score matching addressed confounding. However, the exclusion of individuals with obesity or sarcopenia alone could introduce selection bias.*	*were robust, with stringent QC.However, limited details on imputation accuracy for rare variants and potential batch effects in sequencing reduce the score slightly.*	*standardized pipelines (Illumina NovaSeq), and samples were processed with randomization to avoid batch effects.Blinding and technical consistency across batches were implied.*	*However, potential biases from UK Biobank's volunteer-based recruitment and survivorship bias in older adults were not discussed.*	*provided ample power for exome-wide analyses. Power calculations were implicit in the design.*	*NIE, METAL) and adjustments for multiple testing were well-described. However, exploratory analyses lacked pre-specified hypotheses, and some results were reported selectively.*	*riately adjusted. Multiple testing corrections were rigorously applied. Missing data handling and population stratification control were robust.*	*but details on relatedness checks and haplotype inference were insufficiently described.*	*transparently discussed, and future directions are proposed.*

Ran et al.[32]	*7/7 The study presents a clear hypothesis to identify novel sarcopenia risk genes using whole-exome sequencing (WES) and genome-wide association studies (GWAS).*	*6/7 Whole lean body mass, a proxy for sarcopenia, was measured using DXA (gold standard) in the discovery cohort. However, the replication cohort used bioelectrical impedance analysis (BIA), which has lower precision.*	*7/7 Controls were well-defined.Comparability between discovery (Chinese) and replication (European) cohorts was acknowledged.Detailed selection procedures and replication efforts justify the high score despite population-specific limitations.*	*7/7 WES protocols were robust. Imputation in the UK Biobank used established reference panels.Genotyping platforms and QC steps.were well-documented.*	*6/7 Genotyping methods (e.g., NimbleGen exome array, UK Biobank protocols) are standardized, but the lack of explicit details slightly reduces confidence.*	*6/7 The study acknowledges limitations (e.g., small discovery sample, ethnic homogeneity), but does not fully address potential biases from extreme sampling (e.g., overrepresentation of genetic extremes) or population stratification in replication.*	*7/7 The replication cohort provided sufficient power, compensating for the small discovery sample.. While no a priori power calculation was mentioned, the large replication sample ensured robust validation of suggestive signals.*	*6/7 Analytical methods (linear were appropriate and described.*	*7/7 Confounders were adjusted. Multiple testing correction was applied in discovery. Population stratification was addressed. Missing data handling and sensitivity analyses were rigorous.*	*6/7 HWE and genotype QC were validated, but assumptions about haplotype inference or relatedness were insufficiently detailed.*	*7/7 Conclusions align with results, Limitations (e.g., ethnic diversity, small discovery sample) are transparently discussed, and future directions (functional studies) are proposed.*

ACTN3-Actinin alpha cardiac muscle 3. ACVR1B-Activin A receptor, type 1B. ANCOVA-Analysis of Covariance. ANOVA-Analysis of Variance. BF%-Body Fat. BIA-Bioelectrical Impedance Analysis. CX3CR1-Motif chemokine receptor 1. DNA-Deoxyribonucleic Acid. DXA-Dual-energy X-ray absorptiometry. EASO-European Association for the Study of Obesity. ESPEN-The European Society for Clinical Nutrition and Metabolism .EWGSOP -European Working Group on Sarcopenia in Older People. GTEx-Genotype-Tissue Expression. HGS-Handgrip strength. MTHFR-Methylenetetrahydrofolate reductase. NRF2-Nuclear factor erythroid 2-related factor 2. PCR-RFLP-Polymerase Chain Reaction - Restriction Fragment Length Polymorphism. QC-Quality control.qPCR-quantitative

Polymerase Chain Reaction. RETN-Human Resistin Gene. SMI-Skeletal muscle index.SO-Sarcopenic Obesity. TNF-α-Tumor Necrosis Factor-alpha.TP53-Tumor suppressor protein 5. TWB-Taiwan Biobank. WES-Whole-Exome Sequencing.

Items: 1: Rationale for study, 2: Selection and definition of outcome of interest, 3: Selection and compara-bility of comparison groups, 4: Technical classification of the exposure, 5: Non-technical classification of the exposure, 6: Other sources of bias, 7: Sample size and power, 8: A priori planning of analysis, 9: Statistical methods and control for confounding, 10: Testing of assumptions and inferences for genetic analyses, 11: Appropriateness of inferences drawn from results (33).

Scoring: 1 to 7, 1 being poor,3 being good, 5 being very good and 7 being excellent.

For studies with control groups: Scores ≤35 indicate poor quality studies, >35 and ≤45 indicate studies of moderate quality, and >45 indicate good quality studies.

For studies without control groups: Scores ≤32 indicate poor quality studies, >32 and ≤40 indicate studies of moderate quality, and >40 indicate good quality studies (20).

The table is the final result of the evaluation, which was performed independently by two reviewers, with any discrepancies resolved through discussion or consultation with a third reviewer.

Table S2– Rationale for Risk of Bias Based on Final ROBINS-E Item Scoring

Studies	***Justification of Items***						
	D1	***D2***	***D3***	***D4***	***D5***	***D6***	***D7***
Khanal et al. [22]	*High (Uncontrolled confounding, overestimation of SNP effects.)*	*Low (Genotyping is accurate.)*	*Low (All participants ≥60 years.)*	*Low (Genetic factors are fixed.)*	*Low (99.9% genotyping success)*	*Some concerns (The study employed validated methods for outcome measurement, but concerns arise due to the thresholds used.)*	*Some concerns (Supplementary tables included genotype distributions and assay details, but raw data or full regression results were not provided.)*
Khanal et al.[23]	*Low (The study minimizes confounding bias by adjusting for age and height in its analysis, two critical variables known to influence both sarcopenia and obesity phenotypes).*	*Low (Genotyping is accurate.)*	*Some concerns (The sarcopenic non-obese group had had few participants, limiting statistical power and generalizability for this subgroup.)*	*(Low (Genetic factors are fixed.)*	*Some concerns (While the genotyping success rate was high ,, the study does not explicitly address missing data handling for other variables.)*	*Low (The study employed standardized, validated methods for outcome.)*	*Low (The study reports all relevant outcomes, including significant and non-significant associations, and provides supplementary data online. There is no evidence of selective outcome reporting.)*
Agostini et al. [2]	*Some concerns (Uncontrolled confounding.)*	*Low (Genotyping is accurate.)*	*Low (Standardized case definitions, and transparent recruitment criteria.)*	*Some concerns (While SNPs are fixed, the intervention likely altered miRNA expression, potentially confounding*	*Low (The study reported minimal missing data, with high genotyping success and complete*	*Some concerns (Reliance on functional tests alone may misclassify sarcopenia status. This introduces measurement error, potentially biasing associations between*	*Some concerns (Selective emphasis on positive findings, without full transparency on non-significant SNPs/miRNAs*

				the observed miRNA changes.)	*miRNA measurements in the subgroup undergoing rehabilitation.)*	*SNPs/miRNAs and sarcopenia.)*	*undermines result robustness.)*
Montazeri-Najafabady et al.[24]	*Some concerns (Uncontrolled confounding.)*	*Low (Genotyping is accurate.)*	*Low (Clear inclusion/exclusion criteria were applied. While the sample size is modest and region-specific (limiting generalizability), no systematic selection bias was evident.)*	*Low (Genetic factors are fixed.)*	*Low (The study presents complete data for all variables in the results tables. No explicit mention of missing data or exclusions suggests minimal missingness, and the analysis likely used complete cases without introducing significant bias.)*	*Low (The study employed standardized, validated methods for outcome.)*	*Some concerns (While multiple outcomes were reported, the possibility of selective reporting (e.g., omitting non-significant analyses) cannot be excluded.)*
Ikeda et al.[25]	*Low (The study adjusted for key confounders such as age, gender, BMI, hypertension, diabetes, and dyslipidemia in multivariate analyses.)*	*Low (Genotyping is accurate.)*	*Low (Participants were drawn from a well-defined longitudinal cohort- Toon Genome Study.)*	*Low (Genetic factors are fixed.)*	*Some concerns (The study excluded some participants with incomplete data but did not detail methods for handling missing data.)*	*Low (The study employed standardized, validated methods for outcome.)*	*Low (Pre-specified analyses were reported. Bonferroni correction addressed multiple testing, and supplemental tables provided additional data.)*
Zhang et al. [26]	*Some concerns*	*Low*	*Low*	*Low*	*Low*	*Low*	*Some concerns*

	(Uncontrolled confounding.)	*(Genotyping is accurate.)*	*(Participants were randomly selected from sarcopenia and control groups, with clear exclusion criteria). The sample size was justified, and selection processes were unlikely to introduce bias.)*	*(Genetic factors are fixed.)*	*(The study reported complete data for all analyzed variables, with no indication of missing genotypes or outcome measurements.)*	*(The study employed standardized, validated methods for outcome.)*	*(The study highlights significant associations for polymorphisms but does not mention adjustments for multiple testing, increasing the risk of false-positive findings. Additionally, there is no pre-registered analysis plan, raising concerns about selective reporting.)*
Urzi et al. [1]	*Low (Residual confounding is unlikely to significantly impact results.)*	*Low (Genotyping is accurate.)*	*Some concerns The sarcopenia group was disproportionately smaller than controls., potentially introducing selection bias.*	*Low (Genetic factors are fixed.)*	*Low (The study reported complete data for all variables, with repeated genotyping for missing results. No evidence of missing data influencing conclusions.)*	*Low (The study employed standardized, validated methods for outcome.)*	*Some concerns (The study did not adjust for multiple testing across SNPs, increasing the risk of false-positive findings. There was no pre-registered analysis plan, raising concerns about selective reporting.)*
Bashir et al. [27]	*Some concerns (Uncontrolled confounding.)*	*Low (Genotyping is accurate.)*	*Some concerns (There is no control group of non-sarcopenic individuals in this study. The analysis focused exclusively on a cross-*	*Low (Genetic factors are fixed.)*	*Low (Missing data were minimal and excluded without evidence of bias.)*	*Low (The study employed standardized, validated methods for outcome.)*	*Low (Results align with pre-specified hypotheses, and negative findings (e.g., no muscle mass associations) are reported*

			sectional cohort of individuals with sarcopenia from the LACE trial.)				*transparently, suggesting no selective result reporting.)*
Shrestha et al. [29]	*High (Inability to rule out confounding as an explanation for its findings.)*	*Low (Genotyping is accurate.)*	*Some concerns (While the selection criteria align with the research question, the methodological constraints-early termination, convenience sampling, lack of control, introduce uncertainty about the robustness and generalizability of the findings.The LACE trial was halted prematurely due to poor recruitment.)*	*Low (Genetic factors are fixed.)*	*Low (Missing data were minimal and excluded without evidence of bias.)*	*Low (The study employed standardized, validated methods for outcome.)*	*Some concerns (The study conducted post hoc gender-stratified analyses without pre-specifying these subgroups in the original protocol.)*
Wu et al.[30]	*Some concerns (Uncontrolled confounding.)*	*Low (Genotyping is accurate.)*	*Some concerns (Participants were recruited from a single hospital in Taiwan, limiting generalizability.)*	*Low (Genetic factors are fixed.)*	*Some concerns (The study excluded some participants who failed to complete required measurements, but no details were provided about their characteristics or whether their exclusion introduced bias.)*	*Low (The study employed standardized, validated methods for outcome.)*	*Some concerns (While significant SNPs and genotype scores are reported, the study does not clarify whether multiple testing corrections were applied for genome-wide associations.This increases the risk of selective reporting.)*

Xu et al.[31]	*Some concerns (Uncontrolled confounding.)*	*Low (Genotyping is accurate.)*	*Low (Large sample size ensures robust statistical power.)*	*Low (Genetic factors are fixed.)*	*Low (Missing data were minimal due to the UK Biobank's rigorous phenotyping protocols.)*	*Low (The study employed standardized, validated methods for outcome.)*	*Low (The study pre-specified exome-wide significance thresholds and Bonferroni correction for gene-based tests. Non-significant findings were transparently reported.)*
Ran et al.[32]	*High (Uncontrolled confounding, underestimation of SNP effects.)*	*Low (Genotyping is accurate.)*	*Some concerns (The discovery sample used extreme sampling.)*	*Some concerns (medical interventions or adaptive behaviors based on the studied characteristics: extremely low or high BMD, genetic factors.)*	*Low (The study excluded participants with incomplete measurements but did not explicitly describe methods for handling missing genetic or phenotypic data. However, genetic data completeness in WES and GWAS is typically high (>99%), and no evidence suggests missingness was related to genotype or outcome.)*	*Low (The study employed standardized, validated methods for outcome.)*	*Some concerns (The study reported SNPs with suggestive significance,but did not fully address multiple testing correction for secondary analyses. Non-significant findings were underdiscussed, raising concerns about selective reporting.)*

BIA-Bioelectrical Impedance Analysis. BMD- Bone Mineral Density. DEXA-Dual-Energy X-ray Absorptiometry. GWAS-Genome-Wide Association Study. LACE-Leucine or Angiotensin Converting Enzyme. MiRNAs-MicroRNAs. SMM%-Skeletal Muscle Mass Percentage. SNP-Single Nucleotide Polymorphism. WES-Whole Exome Sequencing.

ROBINS E includes seven domains of bias:
- *Domain 1: Risk of bias due to confounding*
- *Domain 2: Risk of bias arising from measurement of the exposure*
- *Domain 3: Risk of bias in selection of participants into the study (or into the analysis)*
- *Domain 4: Risk of bias due to post-exposure interventions*
- *Domain 5: Risk of bias due to missing data*
- *Domain 6: Risk of bias arising from measurement of the outcome*
- *Domain 7: Risk of bias in selection of the reported result*

The table is the final result of the evaluation, which was performed independently by two reviewers, with any discrepancies resolved through discussion or consultation with a third reviewer.
