# Supplementary material for: Genetic Patterns Related with the Development and Progression of Sarcopenia and Sarcopenic Obesity: A Systematic Review
Source: Medicina (Kaunas). 2025 May 8;61(5):866. doi: 10.3390/medicina61050866 (PMC12113501; doi:10.3390/medicina61050866)
Supplement: Supplementary file 1 [file medicina-61-00866-s001.zip › medicina-3599384- File S2.pdf]

Supplementary Table S3- Functional pathways distribution of the relevant SNPs

Functional pathways	***Gene and SNPs***	***Studies***
Muscle structure, function and atrophy	*ACTN3 rs1815739*	*Khanal et al. [23], Urzi et al. [1]*
	ACVR1B rs10783486,<br>*ACVR1B rs2854464*	*Bashir et al.[27]*
	TP53 (Arg72Pro) rs1042522,<br>*TP53(16-bp Del/Ins) rs17878362*	*Montazeri-Najafabady et al. [24]*
Neurotransmission	*SNAP-25 rs363050*	*Agostini et al.[2]*
Lipid metabolism and adipogenesis	*FTO rs9936385*	*Zhang et al.[26]*
	FTO rs9939609	*Zhang et al.[26], Khanal et al.[22]*
	OSBPL3 rs10282247,<br>*ACER2 rs7022373*	*Wu et al.[30]*
	LYPLAL1-AS1 rs1417066,<br>*LYPLAL1-AS1 rs11205303,*<br>*LYPLAL1-AS1 rs12138590,*<br>*LYPLAL1-AS1 rs13374518,*<br>*LYPLAL1-AS1 rs147871,*<br>*LYPLAL1-AS1 rs188491278,*<br>*LYPLAL1-AS1 rs34360,*<br>*LYPLAL1-AS1 rs34594323,*<br>*LYPLAL1-AS1 rs35706747,*<br>*LYPLAL1-AS1 rs3736533,*<br>*LYPLAL1-AS1 rs4980745,*<br>*LYPLAL1-AS1 rs55687493,*<br>*LYPLAL1-AS1 rs6670062,*<br>*LYPLAL1-AS1 rs76293177*	*Xu et al.[31]*
	SOAT2 rs2272303,<br>*SOAT2 rs11170413,*<br>*SOAT2 rs2272302*	*Ran et al.[32]*
Insulin signaling and glucose metabolism	*RETN (SNP-420) rs1862513,*<br>*RETN (SNP-358) rs3219175*	*Ikeda et al.[25]*
Oxidative stress and inflammation	*MTHFR rs1537516*	*Khanal et al.[23]*
	MTHFR rs1801131	*Khanal et al.[23]*<br>*Urzi et al. [1]*
	NRF2 rs12594956	*Urzi et al. [1]*
	NOS3 rs1799983	*Khanal et al.[22]*
	BDKRB2 rs1799722,<br>*BDKRB2 rs5810761*	*Shrestha et al.[29]*
Hormonal regulation	*ESR1 rs4870044,*<br>*TRHR rs7832552*	*Khanal et al.[22]*
Cell cycle and regeneration	*FZR1 rs740681*	*Ran et al.[32]*
